# Analysis of Complete Nucleotide Sequences of 12 *Gossypium* Chloroplast Genomes: Origin and Evolution of Allotetraploids

**DOI:** 10.1371/journal.pone.0037128

**Published:** 2012-08-02

**Authors:** Qin Xu, Guanjun Xiong, Pengbo Li, Fei He, Yi Huang, Kunbo Wang, Zhaohu Li, Jinping Hua

**Affiliations:** 1 College of Agronomy & Biotechnology, China Agricultural University, Beijing, China; 2 Institute of Cotton, Shanxi Academy of Agricultural Sciences, Yuncheng, China; 3 College of Biological Sciences, China Agricultural University, Beijing, China; 4 Oil Crops Research Institute, Chinese Academy of Agricultural Sciences, Wuhan, China; 5 Cotton Research Institute, Chinese Academy of Agricultural Sciences, Anyang, China; J. Craig Venter Institute, United States of America

## Abstract

**Background:**

Cotton (*Gossypium* spp.) is a model system for the analysis of polyploidization. Although ascertaining the donor species of allotetraploid cotton has been intensively studied, sequence comparison of *Gossypium* chloroplast genomes is still of interest to understand the mechanisms underlining the evolution of *Gossypium* allotetraploids, while it is generally accepted that the parents were A- and D-genome containing species. Here we performed a comparative analysis of 13 *Gossypium* chloroplast genomes, twelve of which are presented here for the first time.

**Methodology/Principal Findings:**

The size of 12 chloroplast genomes under study varied from 159,959 bp to 160,433 bp. The chromosomes were highly similar having >98% sequence identity. They encoded the same set of 112 unique genes which occurred in a uniform order with only slightly different boundary junctions. Divergence due to indels as well as substitutions was examined separately for genome, coding and noncoding sequences. The genome divergence was estimated as 0.374% to 0.583% between allotetraploid species and A-genome, and 0.159% to 0.454% within allotetraploids. Forty protein-coding genes were completely identical at the protein level, and 20 intergenic sequences were completely conserved. The 9 allotetraploids shared 5 insertions and 9 deletions in whole genome, and 7-bp substitutions in protein-coding genes. The phylogenetic tree confirmed a close relationship between allotetraploids and the ancestor of A-genome, and the allotetraploids were divided into four separate groups. Progenitor allotetraploid cotton originated 0.43–0.68 million years ago (MYA).

**Conclusion:**

Despite high degree of conservation between the *Gossypium* chloroplast genomes, sequence variations among species could still be detected. *Gossypium* chloroplast genomes preferred for 5-bp indels and 1–3-bp indels are mainly attributed to the SSR polymorphisms. This study supports that the common ancestor of diploid A-genome species in *Gossypium* is the maternal source of extant allotetraploid species and allotetraploids have a monophyletic origin. *G. hirsutum* AD1 lineages have experienced more sequence variations than other allotetraploids in intergenic regions. The available complete nucleotide sequences of 12 *Gossypium* chloroplast genomes should facilitate studies to uncover the molecular mechanisms of compartmental co-evolution and speciation of *Gossypium* allotetraploids.

## Introduction

Cotton is one of the world's most important crops for producing natural textile fiber. Upland cotton (*Gossypium hirsutum* L.) accounts for more than 90% of production. Three other cultivated species, namely, Sea Island cotton (*G. barbadense* L.), Asian cotton (*G. arboreum* L.) and African cotton (*G. herbaceum* L.), are used in commercial production as well. All the cotton plants and species are classified in taxonomy into the genus *Gossypium* (Malvaceae), a model system for the study of polyploidization in plants [1]. *Gossypium* is comprised of 51 species, including 5 allotetraploid species and 46 diploids [2], [3]. The 5 allotetraploid species are *G. hirsutum* L. AD1, *G. barbadense* L. AD2, *G. tomentosum* Nuttal ex Seemann AD3, *G. mustelinum* Miers ex Watt AD4, and *G. darwinii* Watt AD5, which are further classified into 3 lineages [4], [5]: the *G. mustelinum* AD4 lineage, *G. barbadense* AD2-*G. darwinii* AD5 lineage and *G. hirsutum* AD1-*G. tomentosum* AD3 lineage. The diploid *Gossypium* species are comprised of 8 genome groups, namely, A group through G group and K group. They could cluster into 3 major lineages, corresponding to 3 continental regions: 13 D-genome species from the American continent, 15 species in the African-Asian group (A-, B-, E-, and F group genomes) from African-Asian continent, and another clade of 18 species (C-, G- and K- group genomes) from Australia [4].

The donor species of allotetraploid cottons had been intensively studied [4]–[7], but the origin and evolution of *Gossypium* allotetraploids has remained controversial, with debate over the identity of the progenitor diploid species, the time and mechanism of allopolyploidization. Restriction enzyme digestion of chloroplast DNA (cpDNA) derived from the species of A-, D-, and AD group genomes revealed that chloroplast genomes of 5 tetraploid species were similar to those of *G. arboreum* A2 and *G. herbaceum* A1, leading to speculation that the maternal donor of the allotetraploid originated from A group genome [6]. Because nuclear genomes of *G. herbaceum* A1 and *G. arboreum* A2 differed from A subgenome of allotetraploid cotton through two and three reciprocal translocation, respectively, most researchers considered *G. herbaceum* A1 to be genetically closer than *G. arboreum* A2 to the allotetraploid [3], [7]. Comparative genetic mapping [8], [9] and analysis of DNA sequences [3], [10], [11] indicated that paternal donor of the allotetraploid was similar to extant D group genome in the Americas and the closest living species was *G. raimondii* D5. However, the evolutionary history of *Gossypium* suggested that *G. gossypioides* D6, rather than *G. raimondii* D5, was involved in the origin of allotetraploid cotton [4], [7], [12].

In the absence of a clear fossil record, the divergence of *Gossypium* was always estimated under a molecular clock model [3]. Based on the analysis of *ndhF* sequence and average variation rate in eukaryotes, divergence time between *Gossypium* and its sister-group *Kokia*-*Gossypioides* was calculated to be approximately 12.5 million years ago (MYA) [13]. The divergence of the diploid progenitors was estimated to have occurred around 6.8 MYA using data of some nuclear genes [14]–[16]. Molecular evolutionary data indicated that the *Gossypium* allotetraploid originated during the Pleistocene (0.3 to 2 MYA) [13], [14], [16], predating the evolution of New World humans.

The plastid chromosomes, which have relatively low numbers of genes and high levels of conservation, have provided valuable information to estimate phylogenetic relationship among plants and green algae [17]–[20]. The chloroplast genome sequences of *G. hirsutum* AD1 and *G. barbadense* AD2 published, providing strong reliable evidence for the detailed position of cotton in the Malvales [21], [22]. Though many taxonomic studies have advanced our understanding of *Gossypium*, few data from chloroplast genomes can be used to explore inter- and intra-specific differentiation of *Gossypium*. It is necessary to expand taxon sampling at a high taxonomic level.

To further understand the origin and evolution of *Gossypium* allotetraploids, we sequenced the chloroplast genomes of the following taxa: *G. herbaceum* var. *africanum* A1 and *G. arboreum* A2 from the A-genome lineage; *G. raimondii* D5 and *G. gossypioides* D6 from the D-genome lineage; 2 races of *G. hirsutum* AD1, *G. hirsutum* race *hainansijimian* AD1 and *G. hirsutum* race *lanceolatum* AD1, originating from Hainan Province of China and southern part of United States, respectively; *G. barbadense* AD2 and its 2 local races, *G. barbadense* race *yuanmou* AD2 and *G. barbadense* race *kaiyuan* AD2, from Yunnan Province of China; and 3 wild allotetraploids including *G. tomentosum* AD3, *G. mustelinum* AD4, and *G. darwinii* AD5. Our objectives were to assess dynamic process of the inter- and intra-specific sequence divergence of *Gossypium* chloroplast genomes, and to re-estimate the origin and evolution of *Gossypium* allotetraploids.

## Results

### Size, content and structure of *Gossypium* chloroplast genomes

Twelve *Gossypium* chloroplast genomes were sequenced in this study. Complete DNA sequences were deposited in GenBank under accession numbers HQ325740 through HQ325745 (*G*. *arboreum* A2, *G*. *darwinii* AD5, *G*. *herbaceum* var. *africanum* A1, *G. mustelinum* AD4, *G. raimondii* D5, and *G. tomentosum* AD3, respectively), and HQ901195 through HQ901200 (*G. gossypioides* D6, *G. hirsutum* race *lanceolatum* AD1, *G. hirsutum* race *hainansijimian* AD1, *G. barbadense* race *yuanmou* AD2, *G. barbadense* AD2, and *G. barbadense* race *kaiyuan* AD2, respectively). HQ901199 is a new version of the chloroplast genome sequence of *G. barbadense* AD2.

**Figure 1:**
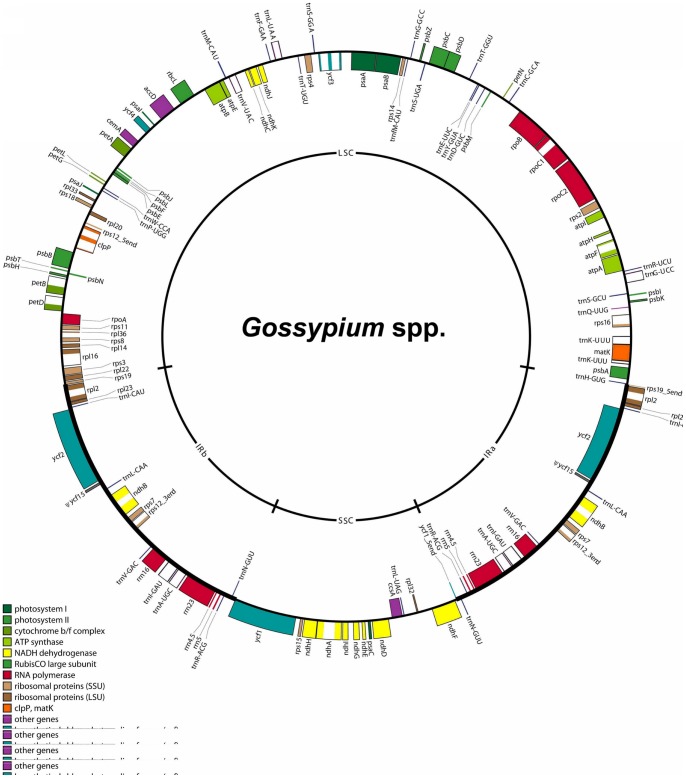
Gene map of *Gossypium* chloroplast genome. The map includes the inverted repeats, IRa and IRb, small (SSC) and large (LSC) single copy regions. Genes exhibited on the inside of the map are transcribed in a clockwise direction, while genes on the outside of the map are transcribed in a reverse order.

The 12 chloroplast genomes were typical circular chromosomes like those of most other higher plants, including the large single copy (LSC), the small single copy (SSC) and 2 IR regions (Figure 1). The whole genome size ranged between 159,959 bp (*G. gossypioides* D6) and 160,433 bp (*G. tomentosum* AD3). The length varied from 88,656 bp (*G. raimondii* D5) to 88,932 bp (*G. tomentosum* AD3) in the LSC region, from 20,004 bp (*G. gossypioides* D6) to 20,285 bp (*G. herbaceum* var. *africanum* A1) in the SSC region, and from 25,576 bp (*G. gossypioides* D6) to 25,650 bp (*G. raimondii* D5) in the IR region (Table 1).

**Table 1:** Size of 13 *Gossypium* chloroplast genomes with the LSC, SSC and IR regions.

Species	Genome (bp)	LSC (bp)	SSC (bp)	IR (bp)
*G. herbaceum* var. *africanum* A1	160,315	88,790	20,285	25,620
*G. arboreum* A2	160,230	88,722	20,274	25,617
*G. hirsutum* AD1	160,301	88,816	20,269	25,608
*G. hirsutum* race *lanceolatum* AD1	160,256	88,799	20,273	25,592
*G. hirsutum* race *hainansijimian* AD1	160,265	88,782	20,279	25,602
*G. barbadense* AD2	160302	88,849	20,267	25,593
*G. barbadense* race *kaiyuan* AD2	160,289	88,836	20,267	25,593
*G. barbadense* race *yuanmou* AD2	160,291	88,838	20,267	25,593
*G. tomentosum* AD3	160,433	88,932	20,271	25,615
*G. mustelinum* AD4	160,313	88,826	20,269	25,609
*G. darwinii* AD5	160,378	88,906	20,266	25,603
*G. raimondii* D5	160,161	88,656	20,205	25,650
*G. gossypioides* D6	159,959	88,803	20,004	25,576

Note: *G. hirsutum* AD1[21].

The corresponding coding sequences accounted for 49.4% of the genome, 48.4%, 71.8%, and 40.8%of LSC, SSC and IR region, respectively. Each genome contained 112 unique functional genes, including 78 protein-coding and 34 RNA-coding genes (Table S1). Eighteen genes were located in IR regions, thus each genome harbored a total of 130 genes. And 34 RNA-coding genes consisted of 30 tRNA genes and 4 rRNA genes, in which 7 tRNA (*trnA_UGC_*, *trnI_CAU_*, *trnI_GAU_*, *trnL_UAA_*, *trnN_GUU_*, *trnR_ACG_*, and *trnV_GAC_*) and 4 rRNA genes (*rrn16*, *rrn23*, *rrn5*, and *rrn4.5*) were located in IR regions. And the other seven genes in IR regions were *ndhB*, *rpl2*, *rps7*, *rps12, rpl23*, *ycf2*, ans *ycf15.* It was found that *accD*, *rpl23*, *ycf1*, and *ycf2* are often absent from plant species [23], but these four genes could be detected among 13 *Gossypium* chloroplast genomes. Similar to other higher plants, 2 pairs of genes had overlapping regions, namely, *atpB-atpE* (3-bp overlap) and *psbC-psbD* (53-bp overlap). All protein-coding genes used a standard plastid/bacterial genetic codon model which started with ATG, except *ndhD* with ACG. To transfer 20 kinds of amino acids, including 61 codons, because 30 tRNA genes were encoded by the chloroplast genome, the remaining 31 tRNAs had to be coded by nuclear genome. Among 112 unique genes, *atpF*, *ndhA*, *ndhB*, *petB*, *petD*, *rpoC1*, *rpl2*, *rpl16*, *rps16*, *trnA-UGC*, *trnG-UCC*, *trnI-GAU*, *trnK-UUU*, *trnL-UAA*, and *trnV-UAC* each had one intron, whereas 2 introns presented in *clpP*, *rps12*, and *ycf3*. Furthermore, *matK* was located within the intron of *trnK-UUU*.

The overall AT content was approximately 62.8%, which was very similar among the 13 *Gossypium* chloroplast genomes. However, different levels of AT content were found across regions, namely, 64.8%, 68.3%, and 57.0% AT content in the LSC, SSC, and IR regions, respectively. In addition protein-coding regions were found to have an AT content of 61.8% whereas non protein-coding regions had an AT content of 68.4%. tRNA and rRNA genes had a slightly lower AT content with 46.9% and 44.5%, respectively. Triple codon analysis demonstrated that the AT content also varied widely with 53.8%, 61.8%, and 69.7% at the first, second, and third positions, respectively (Table S2).

**Figure 2:**
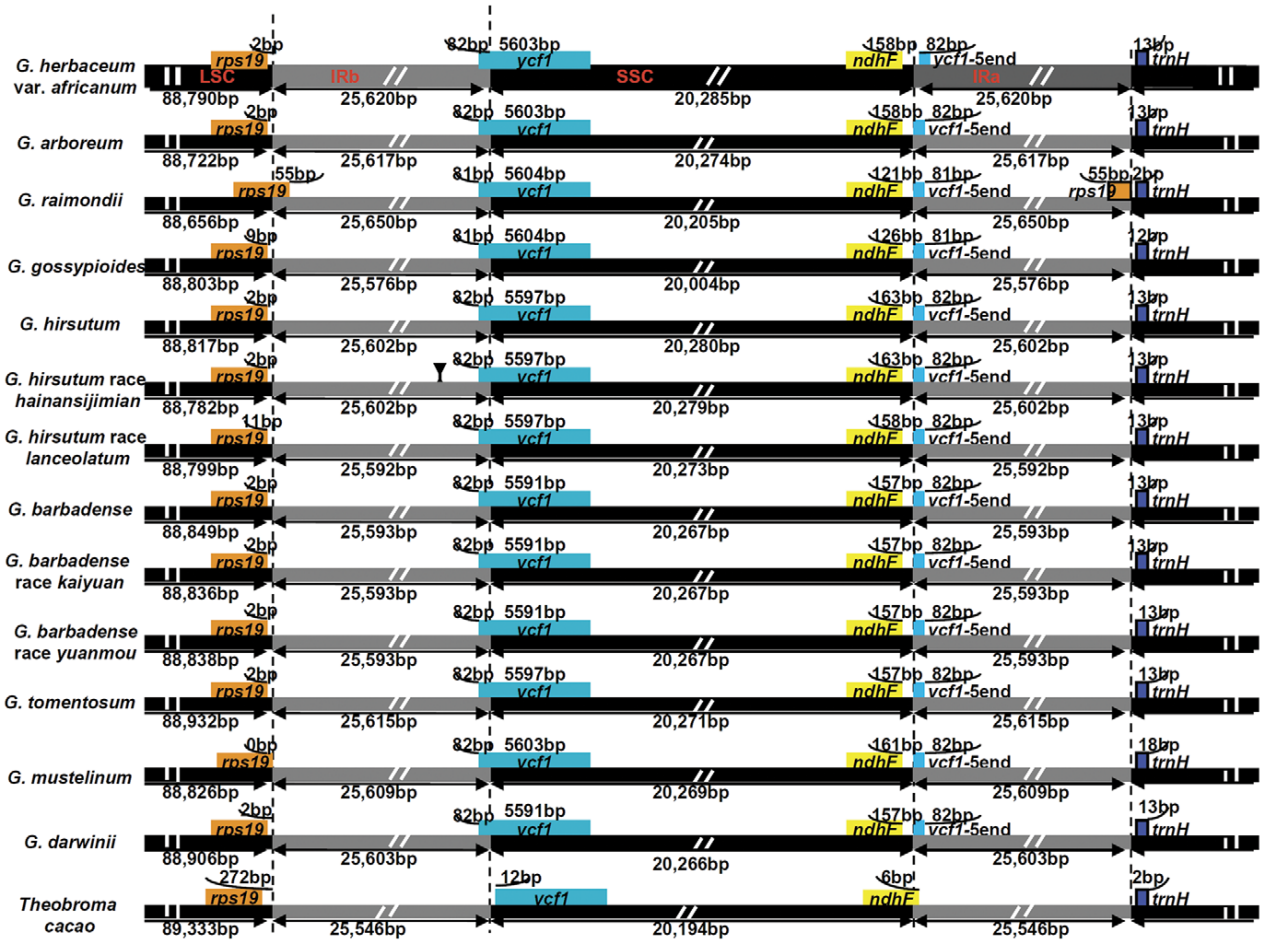
Chloroplast genome IR junctions of 13 *Gossypium* species with *Theobroma cacao* as an outgroup.

Although the overall structure, genome size, gene number, and gene order are well conserved, the junctions between IR and SSC regions are usually different in higher plant chloroplast genomes. Using cacao (*Theobroma cacao* L., Malvaceae), a close relative of *Gossypium*, as outgroup, a slight difference in junction positions was observed among 13 *Gossypium* chloroplast genomes (Figure 2). For example, *rps19* extended into IRb with 55 bp in *G. raimondii* D5, while the whole *rps19* was located within the LSC region in the other 12 *Gossypium* genomes. The distance of *rps19* from the junction of LSC/IRb varied from 0 bp (*G. mustelinum* AD4) to 11 bp (*G. hirsutum* race *lanceolatum* AD1), similar to *Nuphar*, *Ranunculus*, and *Nicotiana*, where distances of 89 bp, 36 bp and 9 bp respectively have been reported. Gene *ycf1* extended slightly into the IR region in *Gossypium* which partly explain why the SSC region in *Gossypium* is larger than that in other plants.

### Genome substitutions and indels between different genomes

Global alignment of the 13 *Gossypium* chloroplast genomes was conducted. The produced alignment spanned over 161.6 kb. In the alignment, the total number of substitutions varied from 6 (AD2-AD2) to 1,000 bp (D6-AD1) (Table 2), and the nucleotide divergence between them was estimated in the range of 0.004%–0.62%. The ratio of transition to transversion (Si/Sv) ranged between 0.2 (AD2–AD2) and 1.2 (A1–A2), with an average value of 0.45 (Table S3). The base substitution type between C and G was significantly less than other types. The total length of substitutions within *G. barbadense* AD2 lineages varied from 6 to13 bp, while that within*iG. hirsutum* AD1 lineages ranged from 25 to 43 bp. Within allotetraploid species, the total substitutions varied from 63 bp (AD2-AD5, with 0.039% divergence) to 175 bp (AD1–AD3, with 0.108% divergence), and from 147 (AD5–A2, with 0.091% divergence) to 231 bp (AD1–A1, with 0.143% divergence) between allotetraploid species and diploid A-genome specie. There were only 11-bp substitutions between A1 and A2, but 180-bp substitutions between D5 and D6. The total length of substitutions between A-genome species and D-genome species varied from 852 (A2–D5) to 916 bp (A1–D6), and the nucleotide divergence between them ranged from 0.527% to 0.567%.

**Table 2:** The substitutions and indel number between any two of 13 *Gossypium* chloroplast genomes.

	Gaf A1	Ga A2	Gh AD1	Ghh AD1	Ghl AD1	Gb AD2	Gbk AD2	Gby AD2	Gt AD3	Gm AD4	Gd AD5	Gr D5	Gg D6
Gaf A1		13	122	134	118	121	121	121	117	110	113	261	265
Ga A2	11		118	129	115	113	113	113	109	107	104	255	259
Gh AD1	224	219		18	22	84	83	82	90	90	82	260	266
Ghh AD1	231	226	25		32	93	91	92	100	94	92	264	269
Ghl AD1	221	216	35	43		81	80	80	85	83	80	255	257
Gb AD2	166	164	152	160	146		5	3	63	85	35	271	278
Gbk AD2	165	164	152	161	146	13		5	62	83	34	270	277
Gby AD2	162	161	149	157	143	6	9		63	84	33	270	277
Gt AD3	162	160	167	175	164	86	85	82		79	48	267	272
Gm AD4	152	147	164	174	168	89	89	86	84		75	260	264
Gd AD5	177	171	151	169	161	66	66	63	104	110		266	274
Gr D5	856	852	940	926	938	873	875	871	862	854	873		70
Gg D6	916	911	985	983	1,000	924	928	923	927	916	906	180	

Note: Gaf, Ga, Gr, Gg, Gh, Ghh, Ghl, Gb, Gby, Gbk, Gt, Gm and Gd indicates G. herbaceum var. africanum A1, G. arboretum A2, G. raimondii D5, G. gossypioides D6, G. hirsutum AD1, G. hirsutum race hainansijimian AD1, G. hirsutum race lanceolatum AD1, G. barbadense AD2, G. barbadense race yuanmou AD2, G. barbadense race kaiyuan AD2, G. tomentosum AD3, G. mustelinum AD4 and G. darwinii AD5, respectively. They are the same hereinafter. The upper triangle shows the number of genome indels and the lower triangle shows the total substitutions of genome.

A comparison of indels between the chloroplast genomes was also carried out (Table 2). The number of indels ranged from 3 to 278, and thelengthier sizes varied from 5 to 1,863 bp (Table S3). The divergence due to indels between them was estimated up to 1.153% (the total length of indels divided by the total length of aligned). The number of indels varied from 33 (AD2-AD5) to 100 (AD1-AD3) and the size varying from 194 (AD2-AD5, with 0.120% divergence) to 559 bp (AD1-AD3, with 0.346% divergence) within allotetraploids. Additionally, the number of indels between allotetraploid species and diploid A-genome species varied from 104 (AD5-A2) to 134 (AD1-A1) with size from 457 (AD4-A2, with 0.283% divergence) to 711 bp (AD1-A1, with 0.440% divergence). Only 13 indels totaling 91 bp were identified between *G. arboreum* A2 and *G. herbaceum* var. *africanum* A1, much less than those within allotetraploid species. Seventy indels totaling 486 bp were identified between *G. gossypioides* D6 and *G. raimondii* D5. The number of indels was more than 255 between diploid D-genome species and other genome species. Taking nucleotide substitutions and indel divergence together, genomic divergence attributed to indels was estimated as 0.091% to 0.143% between allotetraploid species and diploid A-genome, while the nucleotide divergence was estimated as 0.283% to 0.440%. The total sequence divergence was estimated as 0.374% to 0.583%. And total sequence divergence within allotetraploid species was between 0.159% (AD2–AD5) and 0.454% (AD1–AD3). The indels displayed a higher degree of sequence divergence, about 3-fold larger than substitutions. In addition, we observed the correlation between the number of substitutions and the number of indel events (Table 2), and the ratio of nucleotide substitutions events to indels events (S/I) in different pairwise comparison was investigated. Between allotetraploid species and D-genome species, the S/I ratio varied from 3.2 (AD2–D5) to 3.9 (AD1–D5), but 1.3 (AD2–A1) to 1.9 (AD1–A2) between allotetraploid species and A-genome species. And the S/I ratio decreased to about 1.0 within allotetraploid species (AD2–AD4/AD3–AD4). These suggested that the S/I ratio increased when divergence time increased between genomes [24].

**Figure 3:**
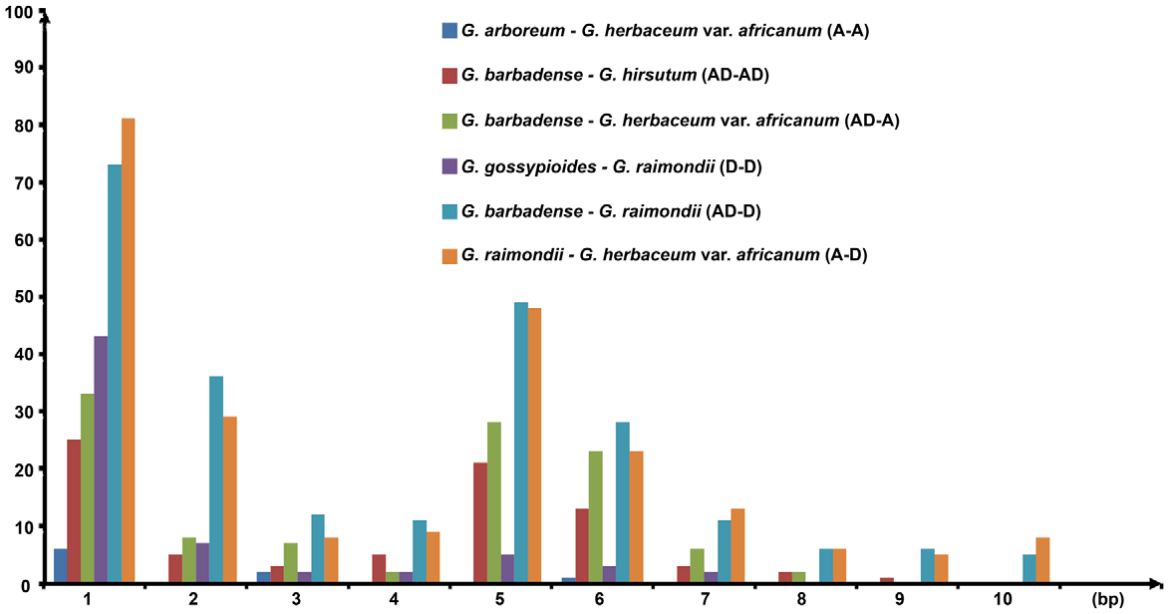
The distribution of indel types in *Gossypium* chloroplast genomes. We pairwise compared the indels of 13 chloroplast genomes. There was a marked tendency for all *Gossypium* species, except for between the 2 A genomes, to fluctuate wherein a point of inflection at the position of 5 bp. In this figure, A, D, and AD genome were represented by *G. herbaceum* var. *africanum* A1, *G. raimondii* D5, and *G. barbadense* AD2, respectively. The *x*-axis indicates the length of indel and *y*-axis represents the number of indels.

The size distribution of indels in our datasets was calculated (Figure 3). The number of short indels (1–10 bp) accounted for more than 90% of the total with more closely related species tending to have a higher proportion of short indels (Table S8A). As expected, single-nucleotide (1-bp) indels were the most common, accounting for approximately 32% of all indels. However, the number of short indels did not decrease with increasing length. The 5-bp indels, accounting for 16% to 24% of all indels, rather than 2-bp indels, were the second most abundant. The number of 5–6-bp indels was significantly more than the number of 3–4-bp indels (Table S8A). The results were consistent across almost all *Gossypium* species by pairwise comparison except for between the 2 A-genomes due to their close relationships.

**Figure 4:**
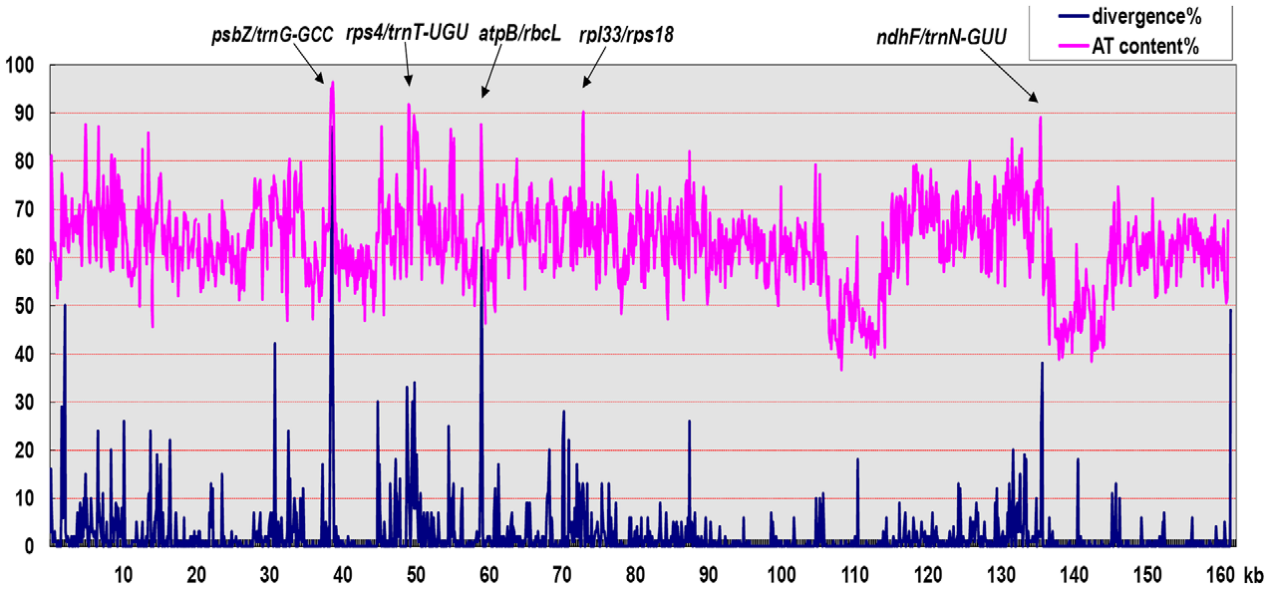
The relationships between sequence divergence and the AT content in *Gossypium* chloroplast genome. AT content was calculated using sliding sequence intervals of 100 bp, while divergence was measured by multiple genome alignment with a step size of 10 bp.

The substitutions and indels were analyzed separately for LSC, SSC, and IR regions (Table S4). The substitution rate (the number of substitutions per kb) was the highest in the SSC region and the lowest in the IR region. For example, 605, 201 and 24 substitutions were detected in the LSC (88.8 kb), SSC (20.3 kb), and IR (25.6 kb) regions between *G. arboreum* A2 and *G. raimondii* D5, with substitution rates of 6.8/kb, 9.9/kb and 0.9/kb, respectively. The substitution rate in SSC was about 11-fold larger than that in IR region. While the highest indel rate was in the LSC region and the lowest was in the IR region. For example, 212, 29 and 9 indels were identified in the LSC, SSC, and IR regions between *G. arboreum* A2 and *G. raimondii* D5, with indel rate (the number of indels per kb) of 2.3/kb, 1.4/kb and 0.4/kb, respectively. Specifically, the indel rate in the LSC region was about 6-fold larger than that in the IR region. Because high sequence divergence was usually accompanied by a high AT content as shown in Figure 4, the higher AT content in SSC region has contributed to a higher substitution rate (Table S2), but the fact that this region has a larger proportion of coding sequences appears to have constrained the indel rate for functional variation. Both the substitution and indel rates confirmed that the IR region was more conserved than single copy regions. And the conserved regions harbored relative lower AT content (Table S2, Figure 4).

### SSR variation in *Gossypium* chloroplast genomes

Simple Sequence Repeats (SSRs) are often used as molecular markers in genetics. SSRs usually have a higher rate of mutation compared to other neutral regions of DNA due to slipped strand mispairing (slippage) during DNA replication on a single DNA strand. A large number of SSR loci were found in *Gossypium* chloroplast genomes. The number of mononucleotide ≥8 bp, dinucleotide ≥8 bp, trinucleotide ≥9 bp, tetranucleotide ≥12 bp, and pentanucleotide ≥ 15 bp loci were counted. The number of SSRs ranged from 315 to 327, and the average rate was 2 SSRs/kb among the 13 genomes. Similarly, the number of mononucleotide ≥10 bp, dinucleotide ≥10 bp, trinucleotide ≥ 12 bp, tetranucleotide ≥16 bp, and pentanucleotide ≥20 bp loci were detected as well. The number of all SSRs varied from 58 to 70, and the average rate was 0.4 SSRs/kb between the 13 genomes (Table 3). Among the different SSRs motifs, they appeared at different frequencies, mononucleotide repeats were most abundant (50.97%–58.09%). The predominant mononucleotide repeats were A or T, which accounted for 95.14% to 97.21% of the mononucleotide repeats among the 13 genomes with a definition mononucleotide ≥8 bp. Most SSRs were located in intergenic spacers, and at least 68 intergenic spacers contained SSR loci among 13 *Gossypium* chloroplast genomes. More than 4 SSR loci were found in *trnK-UUU/rps16*, *atpF/atpH*, *psbZ/trnG-GCC*, *atpH/atpI*, *rpoB/trnC-GCA*, *petN/psbM*, *psaA/ycf3*, *atpB/rbcL*, *psbE/petL*, *ycf4/cemA*, *3*′*-rps12/trnV-GAC*, *petA/psbJ*, *psaJ/rpl33*, and *rpl32/ndhF*. These regions could be utilized as targets for DNA polymorphism analysis.

**Table 3:** Number of SSRs identified in 13 chloroplast genomes.

Species	Mononucleotide		Dinucleotide		Trinucleotide		Tetranucleotide		Pentanucleotide		Total	
	≥8	≥10	≥8	≥10	≥9	≥12	≥12	≥16	≥15	≥20	A	B
*G. herbaceum* var. *africanum* A1	179	51	58	16	72	3	6	0	4	0	319	70
*G. arboreum* A2	181	48	57	16	72	3	6	0	4	0	320	67
*G. hirsutum* AD1	182	44	54	15	71	2	6	0	3	0	316	61
*G. hirsutum* race *hainansijimian* AD1	183	41	54	15	70	2	6	0	2	0	315	58
*G. hirsutum* race *lanceolatum* AD1	182	44	54	16	71	3	6	0	2	0	315	63
*G. barbadense* AD2	184	44	55	15	72	3	5	0	2	0	318	62
*G. barbadense* race *yuanmou* AD2	185	44	55	15	72	3	5	0	2	0	319	62
*G. barbadense* race *kaiyuan* AD2	185	46	55	15	72	3	5	0	2	0	319	64
*G. tomentosum* AD3	182	46	57	16	74	6	5	0	2	0	320	68
*G. mustelinum* AD4	184	43	56	16	72	4	6	0	4	0	322	63
*G. darwinii* AD5	183	44	56	16	72	4	6	0	2	0	319	64
*G. raimondii* D5	186	48	58	12	73	2	7	0	3	0	327	62
*G. gossypioides* D6	190	50	58	12	73	2	6	0	2	0	329	64
Average	184	46	56	15	72	3	6	0	3	0	320	64
Conserved	79	8	36	3	36	0	4	0	0	0	155	11
Conserved rate	0.43	0.18	0.64	0.20	0.50	0	0.69	-	0	-	0.48	0.17

Note: A: The total number of mononucleotide ≥8 bp, dinucleotide ≥8 bp, trinucleotide ≥9 bp, tetranucleotide ≥12 bp, and pentanucleotide ≥15 bp. B: The total number of mononucleotide ≥10 bp, dinucleotide ≥10 bp, trinucleotide ≥12 bp, tetranucleotide ≥16 bp, and pentanucleotide ≥20 bp.

The SSR polymorphism was estimated (Table 3). SSR variation came mainly from the mononucleotide SSRs. Among the 13 chloroplast genomes, 8 out of 46 (17.4%) mononucleotide SSR loci were completely conserved when the mononucleotide repeat unit was defined as ≥10 bp, 71 out of 138 mononucleotide SSR loci (51.4%) with lengths defined as 8 and 9 bp were found to be completely identical. Additionally, the polymorphism of dinucleotide repeats was about 36% in the size group ≥8 bp, which was much lower than that of mononucleotide repeats (57%). Of all analyzed SSR loci, 52% or 83% loci were polymorphic in *Gossypium* chloroplast genomes in the length groups ≥8 bp or ≥10 bp, respectively. These findings indicated that SSR polymorphism is very rich in the cpDNA of *Gossypium* and that the number of polymorphic SSRs would decrease along with SSR length decreasing or SSR motif elongation.

The numbers of polymorphic SSRs were quantified by pairwise comparison among the 13 chloroplast genomes (Table S5). There was wide variation in the number of polymorphic SSRs, ranging from a minimum of 2 loci (between *G. barbadense* race *yuanmou* AD2 and *G. barbadense* race *kaiyuan* AD2) to more than 115 loci (between allotetraploid species and D-genome species). Most of polymorphic SSRs generated 1–3-bp indels between genomes. We observed the relationships between the number of polymorphism SSR loci (Table S5) and the number of 1–3-bp indels (Table S8A), and the total number of 1–3-bp indels was very close to the number of polymorphism SSR loci. We concluded that 1–3-bp indels were mainly attributed to the SSRs polymorphism variation. Using the polymorphic SSR loci we performed the phylogenetic reconstruction. The tree clearly shows that the genomes of A, D, and AD were in different clades and roughly drew an acceptable topology of *Gossypium* (Figure S1).

### Comparison of coding region among sequenced *Gossypium* species

**Figure 5:**
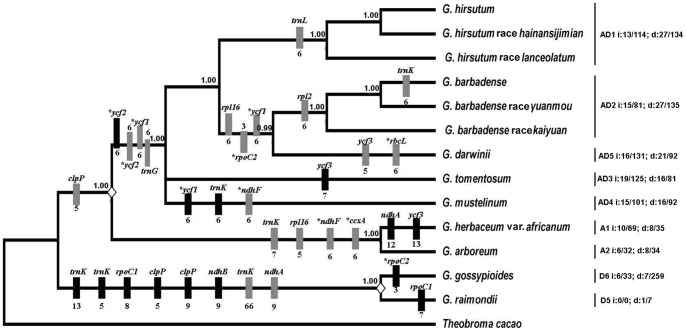
Phylogeny of *Gossypium* species or races. The variable sequences were used to construct a phylogeny of *Gossypium* with *Theobroma cacao* as an outgroup. Bayesian phylogenetic analysis was performed using MrBayes program with GTR + inv + gamma model. The values at nodes represent Bayesian inference posterior probability. On the branch, the insertions (i) and deletions (d) of introns and genes (marked as *) are indicated by black and gray bars. The right tree shows the insertions (i) and deletions (d) of intergenic spacers happened on the branch from the node “<$>\raster="rg1"<$>” to common branches of corresponding genome. Before and after “/” represents the number and length of indels, respectively.

Although chloroplast DNA is, in general, highly conserved in coding region, there are still species differences. Each of the 13 *Gossypium* chloroplast genomes contained 78 unique protein-coding genes, of which 21 genes were completely identical at the DNA level and 19 genes carried only synonymous substitutions (Table S1). Most of these conserved sequences encoded components of the photosystems. The remaining 38 genes harbored amino acid changes between at least one homologous gene pair out of the compared. Seven genes (*atpB*, *atpE*, *psbK*, *rpl2*, *psbM*, *rpl36* and *rps2*) contained only nonsynonymous substitution, and 6 genes had more than 12 mismatches (*rpoB*, *matK*, *rbcL*, *rpoC2, ndhF* and *ycf1* with a maximum mismatch of 12, 13, 16, 21, 28 and 61, respectively). The comparative analysis demonstrated that *ycf1* was the most variable coding region in the chloroplast genome. The nonsynonymous/synonymous substitution rate ratio (dN/dS) of *ycf1* between the A genome and the D genome was significantly greater than 1.0, which could be considered as strong evidence for positive selection. Using *Theobroma* as an outgroup (See Method), 6 genes harbored indels, namely, *rpoC2* (a 6-bp deletion in AD2 and AD5, and a 3-bp insertion in D6), *rbcL* (a 6-bp deletion in AD2), *ycf2* (a 6-bp deletion and a 6-bp insertion in allotetraploids), *ycf1* (a 6-bp deletion in all allotetraploids, a 6-bp deletion in AD2 and AD5, a 6-bp insertion in AD4), *ccsA* (a 6-bp deletion in A1 and A2) and *ndhF* (a 6-bp deletion in AD4, A1 and A2), but no frameshift mutation was detected in these genes. All the insertions and deletions found in the genes were mapped onto the phylogenetic tree (Figure 5). Sixteen genes (*atpI, ccsA, cemA, matK, ndhF, psbK, psbM, rpl16, rpl2, rpoA, rpoB, rpoC1, rpoC2, rps8, ycf1,* and *ycf2*) were diverged among 9 allotetraploid *Gossypium* at the protein level. These genes, having a faster rate of evolution could act be utilized to resolve phylogenetic issues ranging from lower taxonomic ranks to the relationships among genera and species, while those highly conserved genes could be used to help resolve issues at a high taxonomic level.

**Figure 6:**
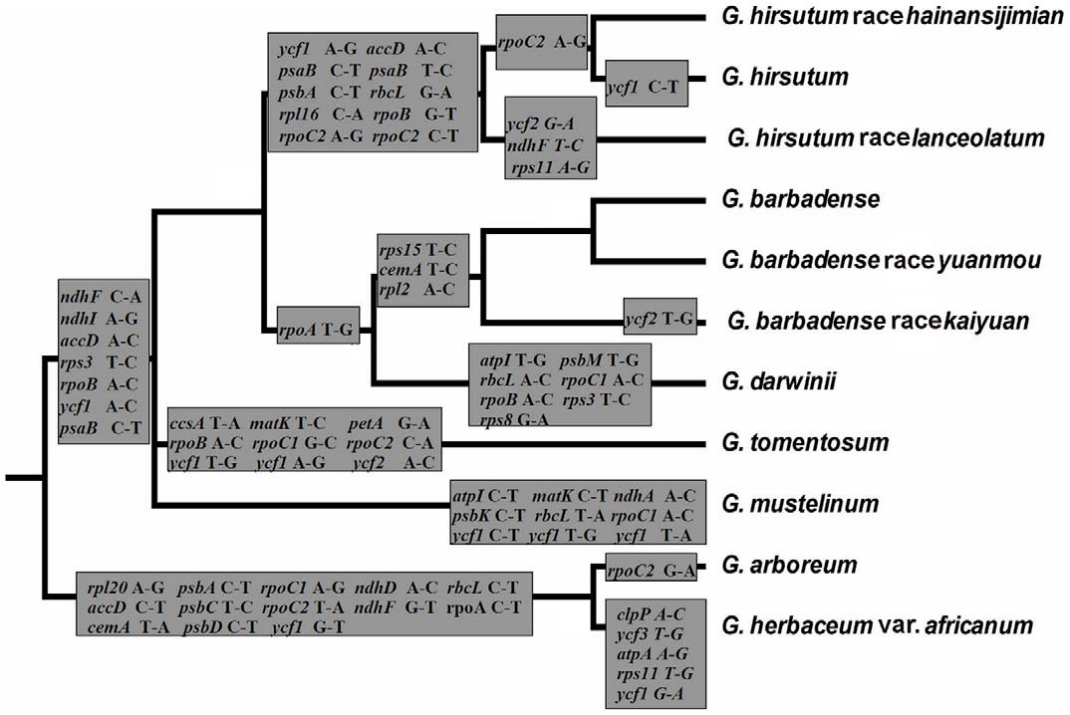
Distributions of the allotetraploid gene substitution. The phylogenetic tree was the subtree of that in Figure 5.

Using *Theobroma* as an outgroup, the substitutions of genes were mapped onto the phylogenetic tree as well (Figure 6). There were 13 genes with a total of 13-bp nucleotide substitutions which occurred in the A genome after allotetraploid formation. Seven genes with a total of 7-bp substitutions had occurred before the split of allotetraploid, while 10 genes, 4 genes, 8 genes, 7 genes, and 8 genes happened after the divergence of allotetraploid in AD1, AD2, AD3, AD4, and AD5, respectively. These suggested that the substitution of *G. hirsutum* AD1 was more than that of its sisters after the divergence of allotetraploid. When AD1 diverged, 1-bp, 2-bp and 3-bp nucleotide substitution occurred in *G. hirsutum* race *hainansijimian*, *G. hirsutum* and *G. hirsutum* race *lanceolatum.* Following the split of A genome, 6-bp and 1-bp substitution occurred in A1 and A2, respectively.

To estimate the functional constraint of each gene group, the nonsynonymous substitution rate (dN), synonymous substitution rate (dS) and dN/dS ratio between *Gossypium* and *Arabidopsis* were calculated (Table 4). The mean dS value of the 13 *Gossypium* genomes varied among gene groups with the highest c-type cytochrome (*ccsA* gene) (0.463±0.0057) and the lowest conserved reading frames (*ycf1* and *ycf2*) (0.0752±0.0004). Mean dN varied more widely than mean dS among the 16 gene groups, such as maturase (0.1911±0.0004) being about 21-fold larger than that of photosystem I (0.0089±0). The dN/dS ratio ranged from a minimum of 0.032±0.0002 for the 5 photosystem I genes, which suggested that these genes were under purifying selection [25], [26], to a maximum of 0.6355±0.0041 for conserved reading frames, which suggested that *ycf1* and *ycf2* experienced rate acceleration [27]. In addition, the dN/dS ratio of gene groups was always positively correlated with AT content. However, a strong positive correlation had not been detected among dN, dS, and dN/dS, which perhaps was due to a statistical bias from similar DNA sequences [28], [29].

**Table 4:** The dS, dN and dN/dS of chloroplast protein-coding gene groups between *Gossypium* and *Arabidopsis.*

Gene group	No. of genes	Length (bp)	AT%	dS	dN	dN/dS
Photosystem I	5	5,979	57.63	0.2794±0.0017	0.0089±0	0.0320±0.0002
Photosystem II	15	5,598	57.92	0.2675±0.0011	0.0113±0.0002	0.0422±0.0007
Cytochrome b/f complex	6	2,364	60.29	0.3797±0.0013	0.0217±0.0002	0.0572±0.0005
ATP synthase	6	4,929	58.35	0.3874±0.0001	0.0314±0.0001	0.0811±0.0004
Assembly/stability of photosystem I	2	1,056	61.11	0.3755±0.0038	0.0342±0	0.0910±0.0010
RuBisCO large subunit	1	1,440	56.03	0.2782±0.0023	0.0371±0.0003	0.1332±0.0016
Ribosomal large subunit protein	8	2,676	59.35	0.2689±0.0031	0.0374±0.0007	0.1392±0.0039
Ribosomal small subunit protein	12	4,998	61.35	0.3142±0.0011	0.0452±0.0002	0.1437±0.0007
NADH genes	11	10,316	64.08	0.3901±0.0007	0.0571±0.0004	0.1464±0.0011
RNA polymerase genes	4	10,423	61.42	0.3630±0.0043	0.0816±0.0007	0.2246±0.0009
Carbon metabolism	1	690	67.20	0.2963±0.0045	0.0827±0.0012	0.2792±0.0064
Proteolysis subunit	1	591	58.19	0.2173±0.0025	0.0613±0.0009	0.2820±0.0050
c-type cytochrome	1	962	66.98	0.4630±0.0057	0.1403±0.0004	0.3029±0.0043
Acetyl-CoA carboxylase subunit	1	1,494	65.38	0.4247±0.0071	0.1801±0.0006	0.4243±0.0073
Maturase	1	1,515	66.27	0.3734±0.0084	0.1911±0.0004	0.5119±0.0105
Conserved reading frames	2	12,570	65.15	0.0752±0.0004	0.0478±0.0002	0.6355±0.0041

Note: Synonymous and nonsynonymous nucleotide substitution rates were estimated according to [69]. Gene group and number of genes were according to Table S1. The data represents mean ± standard error.

Among all the rRNA genes, only *rrn23* in *G. gossypioides* D6 contained a 1-bp substitution. Among all tRNA genes, *trnM-CAU* and *trnP-UGG* in *G. raimondii* D5 and *G. gossypioides* D6, and *trnW-CCA* in *G. herbaceum* var. *africanum* A1 showed a 1-bp substitution. These mutation sites could not be located currently in anti-codon region.

The protein-coding genes were identical at the protein level among the *G. barbadense* lineages as well as between *G. hirsutum* AD1 and *G. hirsutum* race *hainansijimian* AD1 (Table S6). The total length of substitutions ranged from 10 (AD2*–*AD5) to 22 bp (AD1*–*AD3/AD1*–*AD4) within allotetraploid species, accounting for 9.4% (AD1–AD2) to 21.4% (AD3–AD4) of whole genome substitutions divergence (Table S6 and Table 2). Between allotetraploid species and A-genome *Gossypium*, the total length of substitutions varied from 25 (AD2*–*A2) to 38 bp (AD1*–*A1), accounting for 14.2% (AD1–A2) to 22.4% (AD4–A1) of whole genome substitution divergence. Between D-genome species and other species the total length of substitutions varied from 241 (D6*–*AD2) to 259 bp (D5–AD1), accounting for 25.7% (D6–AD1) to 29.8% (D5–A1) of whole genome substitutions (Table S6 and Table 2). And between 2 D-genome species, the total length of substitutions was 60 bp, accounting for 33.3% of whole genome substitution divergence. It suggested a higher proportion of variation rate of the protein-coding sequences after divergence of *G. raimondii* D5 and *G. gossypioides* D6. In addition, the lower proportion of divergence due to protein-coding sequences indicated that the major part of the genomic divergence should be attributed to the non-coding sequences.

### Comparison of non-coding sequences among 13 *Gossypium* chloroplast genomes

Although various functional elements were identified in intergenic spacer regions, e.g., promoter sequences and ribosome-binding sites [30], intergenic spacers were found to be more susceptible to variation than gene sequences. Only 21 out of 128 (16.4%) intergenic spacers were completely identical, however, none of them exceeded 326 bp in length. It suggests that the observed conservation of these intergenic regions can perhaps be attributed to their small size.

The total number of nucleotide substitutions of each pair varied from 5 bp (AD2–AD2) to 673 bp (D6–AD1). And Si/Sv values ranged from 0.20 (AD2–AD3) to 2.00 (AD2–AD2) with an average value of 0.39 (Table S7A), which were less than those of genes. The base substitution between G and T was the most frequent, whereas substition between G and C was the least frequent. The number of indels in each pair ranged from 3 (AD2–AD2) to 229 (D6–AD3/D6–AD2), and the length of a single indel ranged from 1 to 234 bp. The total length of indels varied from 4 (AD2–AD2) to 1642 bp (D6*–*AD5) (Table S7B), and the average length of an indel was from 1.0 to 7.4 bp. Using *Theobroma* as an outgroup, most indels in intergenic spacers of allotetraploids could be classified into insertions or deletions, and the indels that could not be polarized were mostly in the SSR loci. The number of insertions and deletions were plotted onto the phylogenetic tree (Figure 5). There were 4 insertions and 6 deletions which happened before allotetraploids divergence. After the formation of allotetraploid, 27, 27, 16, 16, and 21 deletions and 13, 15, 19, 15, and 16 insertions happened in AD1, AD2, AD3, AD4 and AD5, respectively, These suggested that AD1 and AD2 had more deletions than other allotetraploids, and most allotetraploids, except for *G. tomentosum* AD3, had the biases for deletions since their formation. Additionally, only 8 deletions and 6 insertions occurred on *G. arboreum* A2 after allotetraploid formation. These indicated that allotetraploid had more deletions and insertions than diploid A-genomes.

Seven intergenic spacers, *trnT-UGU/trnL-UAA*, *psbZ/trnG-GCC*, *atpB/rbcL*, *trnK-UUU/matK*, *atpH/atpI*, *rpl32/ndhF*, and *trnL-UAG/rpl32*, were found to be highly variable. The intergenic spacer *psbZ/trnG-GCC* was the most divergent region, containing SSRs, substitutions, and indels, one of which reached 74 bp in length. AT content was typically more than 70% in these intergenic spacers regions, more than the average value of all intergenic spacers, attaining 83% in the intergenic spacer *psbZ/trnG-GCC*. These findings confirmed that the variation, to a large extent, was associated with AT content. A 51-bp insertion was detected in *petN/psbM* of *G. hirsutum* lineages and *G. darwinii* AD5. It indicated that the 51-bp insertion may have occurred independently after the differentiation of AD1 and AD5. In addition, a 234-bp deletion was found in intergenic spacer *trnL-UAG/rpl32* of *G. gossypioides* D6. The deletion was located approximately 820 bp upstream of the *rpl32* start codon. An EST (ES814751) containing *trnL-UAG/rpl32* and *rpl32* was identified from NCBI database, suggesting that the intergenic spacer *trnL-UAG/rpl32* was co-transcribed with *rpl32*. Consequently, the large indel in *trnL-UAG/rpl32* was speculated to regulate *rpl32*.

Indels found in introns were partitioned into insertions and deletions using *Theobroma* as an outgroup (See Method). Ten of the 17 intron sequences harbored indels, including 11 deletions and 11 insertions, and they were: *ndhA* (a 9-bp deletion in D5 and D6, and a 12-bp insertion in A1), *ndhB* (a 6-bp insertion in D5 and D6), *rpl2* (a 5-bp deletion in AD2), *rpl16* (a 5-bp deletion in A1 and A2, and a 5-bp deletion in AD2 and AD5), *rpoC1* (an 8-bp insertion in D5 and D6, and a 7-bp insertion in D5), *trnK-UUU* (a 13-bp and a 5-bp insertion in D5 and D6,a 6-bp deletion in AD2, a 66-bp deletion in D5 and D6, a 6-bp insertion in AD4, and a 7-bp deletion in A1 and A2), *ycf3* (a 5-bp deletion in AD2, a 13-bp insertion in A1, and a 7-bp insertion in AD3), *clpP* (a 5-bp and a 9-bp insertion in D5 and D6, and a 5-bp deletion in A1 and A2), *trnL-UAA* (a 6-bp deletion in AD1), *trnG-GCC* (a 5-bp deletion in allotetraploids). Distributions of the intron insertions and deletions were mapped onto the phylogenetic tree (Figure 5). These data indicated that the *trnK-UUU* intron had more indels than other introns.

### Relationship and origin time of allotetraploid cotton

For evaluation of the phylogenetic relationships of *Gossypium* allotetraploids, 3 groups of sequences, variable protein-coding genes sequences, intron sequences, and variable intergenic sequences, were used to reconstruct the topologies by two independent methods (Bayesian method or maximum-likelihood method), respectively (Figure S2). The 2 topologies from variable protein-coding genes sequences using both maximum-likelihood and Bayesian method showed that allotetraploid divided into four separate branches rapidly after the differentiate. The phylogenetic trees from intron sequences using both maximum-likelihood and Bayesian method, and from variable intergenic spacer sequences using maximum-likelihood, showed that *G. mustelinum* AD4 was the sister to *G. tomentosum* AD3. And the phylogenetic tree from variable intergenic sequences using Bayesian method showed that *G. tomentosum* AD3 was closer than *G. mustelinum* AD4 to *G. hirsutum* lineages. Although the 6 topologies showed slight differences, as a whole, they exhibited great similarity with strong bootstrap or posterior probabilities support, indicating that the constructed phylogenies were highly reliable. In addition, the phylogeny of cotton species or races was reconstructed by using variable sequences with *Theobroma cacao* as an outgroup (Figure 5). The tree suggested a closer relationship of allotetraploid cottons to the 2 A-genome species than to 2 D-genome species. And *G. darwinii* AD5 was the sister to *G. barbadense* lineages, which showed a similar topology as many results [3], [5], [10]. However, in contradiction, the tree showed that the *G. mustelinum* AD4 rather than *G. hirsutum* AD1 was the sister to *G. tomentosum* AD3, which was also described by Wendel et al. [31]. Totally, the phylogenies of *Gossypium* confirmed a close relationship between allotetraploid species and the ancestor of A genome. Also the tree revealed that the split of the common chloroplast genomes of allotetraploid was earlier than that of *G. raimondii* D5 and *G. gossypioides* D6.

Sixty-one common chloroplast protein-coding genes from 2 diploid cotton species, *G. herbaceum* var. *africanum* A1 and *G. raimondii* D5, and 11 other plant species were used to reconstruct the phylogeny (Figure S3). The average length of the 61 concatenated sequences was 42,746 bp. Monocots and eudicots were classified into 2 distinct groups, and the Malvales and Brassicales had a close relationship. Each clade was supported with high bootstrap values. Divergence time between monocots and eudicots was evaluated as 145 MYA in previous studies [32]–[34]. It was defined as the start point to estimate the divergence time within the cotton genus. The divergence time of nodes B, C, and D were estimated to be 127.9, 94.6, and 3.4 MYA, respectively. This suggested that *G. herbaceum* var. *africanum* A1 and *G. raimondii* D5 diverged about 3.4 MYA, which could be used as a reference point.

A-genome and D-genome type cytoplasms represent the 2 large clades of *Gossypium*, which in turn represent the diversification of *Gossypium*. Nucleotide substitutions of 61 protein-coding genes were used to estimate the synonymous rate (rs) and nonsynonymous substitution rate (ra) between *G. herbaceum* var. *africanum* A1 and *G. raimondii* D5. The results showed that rs and ra were 1.162×10^−9^ and 0.206×10^−9^, respectively. The divergence time between *G. herbaceum* var. *africanum* A1 and *G. raimondii* D5 was estimated based on Ka and Ks values of 78 protein-coding genes. Because the calculation using the Ks value was more similar to the branch length method (Table 5), the Ks value was adopted to calculate the divergence time of cotton species. Differentiation between A-genome and D-genome cytoplasms was estimated to have occurred around 3.89 MYA. Allotetraploid species originated 0.43 (AD2–A2) to 0.68 (AD1–A1) MYA and initially diverged 0.38 (AD1–AD3) MYA, *G. darwinii* AD5 diverged from *G. barbadense* lineages about 0.09 MYA. The split of the 2 A-genome species occurred approximately 0.13 MYA, following the formation of allotetraploid cottons. Consequently, it was the ancestor of A-genome *Gossypium* species that was the maternal source of allotetraploid species. The differentiation of chloroplast genomes between *G. raimondii* D5 and *G. gossypioides* D6 was estimated to have occurred around 0.77 MYA, earlier than the advent of allotetraploid cottons.

**Table 5:** Evolutionary distance (MY) between any two of 13 *Gossypium* calculated by chloroplast protein-coding gene.

	Gaf A1	Ga A2	Gh AD1	Ghh AD1	Ghl AD1	Gb AD2	Gbk AD2	Gby AD2	Gt AD3	Gm AD4	Gd AD5	Gr D5	Gg D6
Gaf A1		0.04	0.13	0.13	0.13	0.13	0.13	0.13	0.13	0.13	0.13	0.34	0.34
Ga A2	0.13		0.13	0.13	0.13	0.13	0.13	0.13	0.13	0.13	0.13	0.34	0.34
Gh AD1	0.68	0.60		0.04	0.04	0.09	0.09	0.09	0.13	0.09	0.09	0.34	0.34
Ghh AD1	0.64	0.56	0.04		0.04	0.09	0.09	0.09	0.09	0.09	0.09	0.34	0.34
Ghl AD1	0.68	0.60	0.04	0.04		0.09	0.09	0.09	0.13	0.09	0.09	0.34	0.34
Gb AD2	0.47	0.43	0.30	0.26	0.30		0.04	0	0.09	0.09	0.04	0.34	0.34
Gbk AD2	0.51	0.47	0.30	0.30	0.30	0.04		0.04	0.09	0.09	0.04	0.34	0.34
Gby AD2	0.47	0.43	0.30	0.26	0.30	0	0.04		0.09	0.09	0.04	0.34	0.34
Gt AD3	0.60	0.56	0.38	0.38	0.38	0.21	0.26	0.21		0.09	0.09	0.34	0.34
Gm AD4	0.51	0.47	0.30	0.30	0.30	0.13	0.17	0.13	0.26		0.09	0.34	0.34
Gd AD5	0.51	0.47	0.30	0.30	0.30	0.09	0.13	0.09	0.26	0.21		0.34	0.34
Gr D5	3.89	3.89	3.89	3.85	3.89	3.68	3.72	3.68	3.80	3.72	3.72		0.17
Gg D6	3.89	3.89	3.89	3.85	3.89	3.68	3.72	3.68	3.80	3.72	3.72	0.77	

Note: The lower triangle shows the divergence time (million years) estimated by 78 chloroplast protein-coding genes, and the upper triangle shows the ± standard error.

## Discussion

In this study we present the complete sequences of 12 *Gossypium* chloroplast genomes, including 2 A-genome lineages (*G. herbaceum* var. *africanum* A1 and *G. arboreum* A2), 2 D-genome lineages (*G. raimondii* D5 and *G. gossypioides* D6), and 8 allotetraploid cottons (*G. hirsutum* race *hainansijimian* AD1, *G. hirsutum* race *lanceolatum* AD1, *G. barbadense* AD2, *G. barbadense* race *yuanmou* AD2, *G. barbadense* race *kaiyuan* AD2, *G. tomentosum* AD3, *G. mustelinum* AD4 and *G. darwinii* AD5). The data obtained has permitted an evaluation of the relationship and evolution of *Gossypium* allotetraploids. The results indicate that although the *Gossypium* chloroplast genome is highly conserved, sequence diversification is evident. Comparison of 13 closely related chloroplast genomes including previously published and newly sequenced genomes provided a potentially powerful means to evaluate the relationship and evolution of *Gossypium* allotetraploids. In this study, most indels in both genes and intergenic spacers could be polarized into insertions or deletions, and evolutionary point at which they arose could be estimated using *Theobroma cacao* as an outgroup. It is interesting to find that 5-bp indels appear to be preferred in the *Gossypium* chloroplast genomes. It supported that the ancestor of A-genome *Gossypium* species was the maternal source of allotetraploid species and allotetraploids evolved from a common ancestor. The progenitor allotetraploid *Gossypium* originated approximately 0.43 to 0.68 MYA and initially diverged about 0.38 MYA.

### Conservation of *Gossypium* chloroplast genome

Each of the 13 genomes were found to encode the same set of 112 unique genes in a uniform order with a sequence identity >98%. Thus the data demonstrates that the *Gossypium* chloroplast genome is highly conserved. Most genes did not show any variation among the 13 *Gossypium* chloroplast genomes. There was little sequence variation between the genomes of *G. hirsutum* AD1 and its 2 races as well as between *G. barbadense* AD2 and its 2 races (Table 2). And there were only 13 indels and 11 substitutions between *G. herbaceum* var. *africanum* A1 and *G. arboreum* A2. Although great phenotypic variation are observed among them, it is difficult to separate them using chloroplast markers. There were 70 indel events and 180 substitution events between diploid D5 and D6 genomes, while only 13 indels events and 11 substitution events occurred between A1 and A2 genomes. In our study, we seldom used the data of previously sequenced chloroplast genome of *G. barbadense* AD2 [22] (marked as *G. barbadense 2006*, hereinafter), and the chloroplast genomes of *G. barbadense* AD2 lineages were re-sequenced. By comparing the whole genome sequences of *G. barbadense* cv. Zhonghai 7 and *G. barbadense 2006*, we detected 284-bp substitutions and 103-bp indels, larger than that between allotetraploid and A-genome species.

### Indels of *Gossypium* chloroplast genome

Many recent evolutionary variations, notably indels, were found by comparing 13 chloroplast genomes. These indel events were mainly attributed to the perfect or near-perfect repetition of an adjacent sequence, probably caused by slipped-strand mis-pairing in DNA replication [35]. Indels are thought to be a major driving force in sequence evolution [16], [36]. The number of indels decrease rapidly with increasing indel length [37]–[39]. However, the 5–6-bp indels, which was caused by adjacent 5–6-bp motif duplication or loss, are the second most common type in our study (Figure 3 and Table S8A). We also compare intraspecies or inter-subspecies of *Acorus*, *Aethionema*, *Nicotiana*, *Oryza*, *Oenothera*, *Populas*, and *Solanaceae* (Table S8B). Only *Oenothera* has similar, but much weaker results as *Gossypium*. The common ground between *Gossypium* and other species is the relatively few indels of 4-bp in length. It is necessary to further investigate whether a preference for 5-bp indels has happened in the nuclear genome of *Gossypium*.

The S/I ratio increased when divergence time increased between genomes. When we explained the increasing of genome S/I ratio as divergence time increased, one aspect may attribute to systematic underestimation of indels in more distantly related species [24]. The other reason may be that the indels scarcely appeared in chloroplast coding regions where substitutions tend to accumulate as divergence time increased. Nevertheless, we also observed that the S/I ratio increased as divergence time increased in just noncoding region (Table S7B).

Multiple closely related genomes were always required to distinguish insertions from deletions [40]. In our study, the number of all indels was counted by pairwise comparison, and most indels could be polarized into insertions and deletions using *Theobroma cacao* as outgroup (See Method). Insertions and deletions were mapped onto the phylogenetic tree (Figure 5). As shown in Figure 5, after the divergence of allotetraploids, the number of deletions was approximately 2-fold larger than that of insertions in the intergenic spacers of AD1 and AD2 genomes, and only deletions were detected in introns and coding sequences of AD1 and AD2 genomes. Although each genome experienced more deletions than insertions to various extents, the average length of single insertion was longer than that of deletion. During the period before the formation of allotetraploid and after the divergence of diploid A genome and D genome (data not shown in the Figure 5), 27 deletions (108 bp) and 19 insertions (179 bp) happened during the stage of 3.2 million years, while 18 deletions (132 bp) and 32 insertions (326 bp) happened before the split of diploid D5 and D6 genomes during the stage of 3.12 million years in whole genomes. Based on our evaluated divergence time and the number of indels, the indel ratio was standardized to the number of indels per genome per million years. These results showed that insertion ratio as well as deletion ratio was incongruent in the 2 branches after the split of A genome and D genomes, but the indel ratio converged to a fixed level for a substantial period of time.

### Origin and evolution of *Gossypium*

A global molecular clock among species typically does not exist, and the rate of genome variation is known to change among inter-species specifically over time [45]. Because branch length method does not assume a strict clock, it is a more precise tool for estimating divergence time [32], [46]. Thus the branch length method was used to estimate the divergence time between *Gossypium* and other plant species. However, a likelihood ratio test of substitution rate constancy across species indicated that the protein-coding sequences of *Gossypium* chloroplast genomes had an equal evolutionary rate (P = 0.947). This suggested that a constant molecular clock method could be appropriate to date the divergence time of *Gossypium*. The divergence time between *Gossypium* and other dicotyledons based on our datasets was consistent with previous reports [33], [47]. Little incongruence on substitution rate among the 13 chloroplast genomes was observed in this experiment with the molecular clock method, and the estimated synonymous substitution rate (*r* = 1.162×10^−9^) was very close to previous estimates (*r* = 1.242×10^−9^) [48].

The synonymous substitution rate of the chloroplast genome was considerably different from that of the nuclear genome. The synonymous substitution rate for nuclear genes in plants was from 2.6×10^−9^ to 1.5×10^−8^ [49], which was 2 to 13 times higher than that in the chloroplast genome. Based on this synonymous substitution rate and using various nuclear gene sequence data, the formation of allotetraploids was estimated to have occurred approximately 1-2 MYA. In this study, the divergence time between A-genome species and allotetraploid cotton was estimated to be 0.43 to 0.68 MYA, which is very similar to previous reports [6], [14], [16]. Analysis of the *ndhF* sequence by Seelanan et al. [13] (assuming an average mutation rate of cpDNA genes *r* = 5×10^−10^), indicated that the divergence time between *G. herbaceum* A1 and *G. hirsutum* AD1 was arround 2 MYA much earlier than our estimation. These incongruent findings could be explained by that the substitution rate of *ndhF* sequence was the most rapid, which would not reflect the average substitution rate (*r* = 1.162×10^−9^).

Previous little was known regarding the divergence time of *G. arboreum* A2 and *G. herbaceum* A1 [3]. In this study, the data has shown that there is little variation between *G. herbaceum* var. *africanum* A1 and *G. arboreum* A2, only 13 indels events and 11 substitution events, suggesting that their divergence occurred at a similar time and thus inclusion of *G. herbaceum* A1 was probably merited. The lower level of sequence variation between *G. herbaceum* var. *africanum* A1 and *G. arboreum* A2 could possibly result in inaccurate estimations of the divergence time. However it is quite plausible that *G. herbaceum* var. *africanum* A1 and *G. arboreum* A2 diverged after the formation of allotetraploid taxa. In view of the highly conserved chloroplast genome sequences, it also seems likely that *G. herbaceum* A1 and *G. arboreum* A2 diverged after the formation of allotetraploids.

Although phylogenic analysis of *Gossypium* suggested that the split of the common chloroplast genomes of allotetraploid occurred slightly earlier than that of *G. raimondii* D5 and *G. gossypioides* D6, it could not suggest that the ancestor of the 2 D-genome species, rather than its offspring, were the close relatives of allotetraploids because of the intergenomic introgression between *G. raimondii* D5 and *G. gossypioides* D6 [3]. However, the time of divergence between *G. gossypioides* D6 and *G. raimondii* D5 was estimated to be slightly earlier than the formation of allotetraploids based on protein coding gene sequences. Altogether, it suggested that divergence of the chloroplast genomes between *G. gossypioides* D6 and *G. raimondii* D5 occurred at a time close to that of allotetraploid formation.

**Figure 7:**
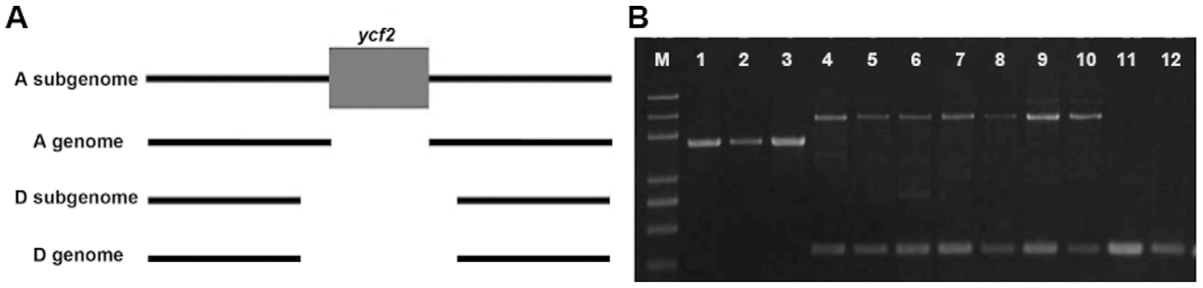
The transfer of the *ycf2* fragment to the nucleus. A: Schematic view of the transfer of cpDNA *ycf2* fragment; B: PCR amplification of the nuclear genome flanking the *ycf2* insertion. M: D2000 plus; 1–12: *G. herbaceum* A1, *G. herbaceum* var. *africanum* A1, *G. arboreum* A2, *G. hirsutum* AD1, *G. hirsutum* race *hainansijimian* AD1, *G. barbadense* AD2, *G. barbadense* race *yuanmou* AD2, *G. barbadense* race *kaiyuan* AD2, *G. mustelinum* AD4, *G. darwinii* AD5, *G. raimondii* D5, and *G. gossypioides* D6, respectively.

The allotetraploids shared 14 indels and 7-bp nucleotide substitutions in protein-coding genes (Figure 5 and Figure 6). The presence of unique indels and substitutions among all allotetraploids indicated that they possibly evolved from a common ancestor. Many studies had confirmed that cpDNA could frequently transfer to nuclear and mitochondrial genomes [41]–[43]. Although the information from chloroplast genomes could provide little evidence about the paternal donor of allotetraploids, the transfer of cpDNA to the nucleus could be helpful to recognize it. Grover et al. [44] found that a *ycf2* fragment was inserted into the A_T_ genome (A genome in tetraploids) but not into D_T_ genome (D genome in tetraploids) of *G. hirsutum* AD1. Here additional *Gossypium* species were investigated for insertion of the *ycf2* fragment which was found to be present in the A_T_ genome of *G. hirsutum* AD1, *G. hirsutum* race *hainansijimian* AD1, *G. barbadense* AD2, *G. barbadense* race *yuanmou* AD2, *G. barbadense* race *kaiyuan* AD2, *G. mustelinum* AD4 and *G. darwinii* AD5, but was not detected in *G. herbaceum* A1, *G. herbaceum* var. *africanum* A1 and *G. arboreum* A2, *G. raimondii* D5 and *G. gossypioides* D6, or the D_T_ genome. Furthermore, it was verified in *G. robinsonii* from C genome, *G. somalense* from E genome, and *G. longicalyx* from F genome as well, but none of them had this *ycf2* fragment in nuclear genomes (Figure 7). The cpDNA *ycf2* fragment was not found in nuclear genome of verified diploid cottons, including A, C, D, E, and F genomes, which suggested that the *ycf2* fragment probably did not exist in the nucleus of the ancestor of A-genome species. So the ancestral allotetraploid lineage from inter-genomic hybridization initially lacked the *ycf2* gene fragment in its nuclear genome. We could speculate that transfer of the *ycf2* fragment occurred earlier than the divergence of allotetraploids, but later than tetraploid formation. Altogether, extant allotetraploid cottons probably share the same ancestor containing the cpDNA-derived *ycf2* fragment from in its nuclear genome. It appears reasonable to infer that the probable paternal donor like the maternal donor, was monophyletic, though details regarding this are still vague.

### Different divergence rate of allotetraploids

Indels play an important role in estimating genome evolution rate [16]. Indel ratio was standardized to the number of indels between genomes per million years (Table S9). Because indels scarcely appeared in chloroplast coding regions, it actually revealed the noncoding indel rates. Indel ratio between any two of allotetraploids varied from 92 to 323, while that was 33 to 34 between diploid A-genome species and diploid D-genome species and even much lower within A genome or D genome. This suggested that indel ratio was inconsistent within *Gossypium* species, and the chloroplast genomes of allotetraploids experienced higher indel ratio than those of diploids. In comparison with a relatively constant indel ratio of *Gossypium* species before the formation of allotetraploids (See above), the higher indel ratio of allotetraploids might be a result of a higher degree of evolutionary variability following the polyploidization [15], [16].

Whole genomes pairwise comparison among allotetraploids showed sequence variation, notably for *G. hirsutum* lineages being more variable (Table 2). So we tested the relative divergence rate between allotetraploid species and A-genome species with D-genome species as outgroup by Tajima's relative rate test [50]. According to the monophyletic theory, all allotetraploids should share a similar genetic distance to the A-genome group. Expectedly, the relative rates of 9 allotetraploid genomes showed no significant difference at the concatenated protein-coding gene level. However, a faster rate of variation was detected in *G. hirsutum* AD1 and its 2 races relative to other allotetraploids at the whole genome level (Table 6). It is probable that the incongruency of the rate was mainly determined by the presence of intergenic spacers.

The indel rate of allotetraploid species was also estimated. All allotetraploids had very similar indel rates at the protein-coding gene level compared to A-genome group, but *G. hirsutum* AD1 lineages experienced more indel events than other allotetraploids in intergenic spacers. Altogether, the intergenic spacers of *G. hirsutum* AD1 and its 2 races, *G. hirsutum* race *hainansijimian* AD1 and *G. hirsutum* race *lanceolatum* AD1, experienced faster divergence rates than other allotetraploids.

**Table 6:** Tajima's relative rate test of chloroplast protein-coding genes and whole genome between *Gossypium* allotetraploid and A-genome diploid with *G. raimondii* D5 as an outgroup.

	Protein-coding genes		Whole genome	
Species	*G. herbaceum* var. *africanum* A1	*G. arboreum* A2	*G. herbaceum* var. *africanum* A1	*G. arboreum* A2
*G. hirsutum* AD1	0.6219	0.3841	0.0052**	0.0024**
*G. hirsutum* race *hainansijimian* AD1	0.7389	0.4795	0.0032**	0.0014**
*G. hirsutum* race *lanceolatum* AD1	0.5164	0.3035	0.0184*	0.0094**
*G. barbadense* AD2	0.3532	0.5485	0.6310	0.4640
*G. barbadense* race *yuanmou* AD2	0.3532	0.5485	0.8711	0.6805
*G. barbadense* race *kaiyuan* AD2	0.4652	0.6949	0.6310	0.4639
*G. tomentosum* AD3	1	0.7150	0.5164	0.6801
*G. mustelinum* AD4	1	0.7150	0.0707	0.1144
*G. darwinii* AD5	0.8618	0.8527	0.2143	0.1343

Note: *p<0.05, **p<0.01.

### Conclusions

The data presented here represents a thorough analysis of the Gossypium chloroplast genomes utilizing both comparative genomics and phylogenetic analyse. The availability of complete nucleotide sequences of 13 *Gossypium* chloroplast genomes provides an opportunity to study the mechanisms under the origin and evolution of *Gossypium* allotetraploids. Although many valuable taxonomic studies have advanced our understanding of *Gossypium*, this is the first effort using such an extensive sample of chloroplast genomes to further understanding inter- and intra-specific differentiation of *Gossypium*. By comparing 13 *Gossypium* chloroplast genomes, species sequence variations have been detected, including substitutions and indels. *Gossypium* chloroplast genomes have a preference for 5–6-bp indels caused by adjacent 5–6-bp motif duplication or loss, whereas 1–3-bp indels are mainly attributed to the SSR polymorphisms. The genome S/I ratio increased as divergence time increased because the indels scarcely appeared in chloroplast coding regions where substitutions tended to accumulate as divergence time increased. This study offers support to the theory that the ancestor of A-genome *Gossypium* species is the maternal source of allotetraploid species. In addition, the transfer of cpDNA *ycf2* to the nuclear genomes shows that the paternal donor is probably monophyletic. The differences of indel ratio among genomes reveal that allotetraploids experienced faster sequence gain or loss than diploids. In addition, our data show that the intergenic spacers of *G. hirsutum* lineages have experienced faster divergence rates than other allotetraploids. The availability of complete nucleotide sequences of 12 *Gossypium* chloroplast genomes should facilitate research to uncover the molecular mechanism of evolution of *Gossypium* allotetraploids.

All data obtained in this study but not shown in the manuscript are available upon request.

## Materials and Methods

### Plant taxa

Species or races chosen for this study were based on closely related lineages for *Gossypium* allotetraploid. Three wild allotetraploid species, *G. tomentosum* AD3, *G. mustelinum* AD4 and *G. darwinii* Watt AD5, as well as each 2 races of *G. hirsutum* (*G. hirsutum* race *hainansijimian* AD1 and *G. hirsutum* race *lanceolatum* AD1) and *G. barbadense* (*G. barbadense* race *yuanmou* AD2 and *G. barbadense* race *kaiyuan* AD2), were adopted for more datasets representing allopolyploid cottons. *G. hirsutum* AD1, *G. hirsutum* race *hainansijimian* AD1 and *G. hirsutum* race *lanceolatum* AD1 were considered as *G. hirsutum* lineages, while *G. barbadense* AD2, *G. barbadense* race *yuanmou* AD2 and *G. barbadense* race *kaiyuan* AD2 were treated as *G. barbadense* lineages. *G. raimondii* D5 and *G. gossypioides* D6 were chosen since they are the best candidates as living models of the D-genome donor. The 2 A-genome lineages (*G. arboreum* A2 and *G. herbaceum* var. *africanum* A1) were indispensable for the present purpose. In addition, unreliable phylogeny of *G. barbadense* AD2 was found based on published datasets, so *G. barbadense* AD2 cultivar ‘Zhonghai 7’ (The inbred line originated from *G. barbadense* AD2 cultivar ‘Xinhai 7’ bred in Xinjiang, China) was adopted to represent the important clade of allopolyploid cottons.

All the materials were collected from Cotton Research Institute, Chinese Academy of Agricultural Sciences, Anyang, Henan, China.

### Chloroplast genome sequencing

CpDNA was prepared from fresh leaves following a previous published protocol [51]. CpDNA of *G. raimondii* D5 was sheared into random fragments of 35 kb to 50 kb, which were subsequently cloned into a pCC1FOS vector to generate a Fosmid library. Using 6 cpDNA markers (Table S10), 6 clones were screened accounting for the whole genome; 4 of 6 clones were then shotgun sequenced using an ABI 3730 sequencer (Applied Biosystems). Sequence coverage of each clone was more than 6-fold. The other 2 clones were sequenced using a general PCR method. The primers were designed according to chloroplast sequences of *G. hirsutum* AD1 [21].

The cpDNA of another 11 cotton species or races were sequenced with Solexa sequencing technology [52]. Each inverted repeat (IR) region was identified by 2 long PCR products (Table S10), each approximately 13 kb in length, and was purified for sequencing separately with Solexa.

Sequences were assembled with programs Phrap (http://www.phrap.com) and Velvet (hash length  = 21, cov_cutoff  = 30) [53]. Chloroplast genes were annotated using an online DOGMA tool (http://dogma.ccbb.utexas.edu/) [54] with *G. hirsutum* AD1 as a guide. Genome maps were drawn with OGDRAW [55].

### Sequence divergence analyses

The chloroplast genome sequences of 12 *Gossypium* species or races were assembled by the authors, and those of *G. hirsutum* AD1 and *G. barbadense* 2006 AD2 were downloaded from GenBank, however the *G. barbadense* AD2 chloroplast genome was analyzed mainly based on our re-sequencing data. Sequence alignment including coding sequences, introns, and intergenic spacers were carried out by CLUSTALW. The chloroplast simple sequence repeats (SSRs) were searched by SSR Extractor under the repeat length definition ≥8 bp (http://www.aridolan.com/ssr/ssr.aspx).

### The transfer of *ycf2* to nuclear genome

For detecting the transfer of cpDNA to nuclear genome, we aligned the sequence of cpDNA across the genus using sliding sequence with a step size of 1000 bp. Finally, a *ycf2* fragment was found in a bacterial artificial chromosome (BAC) of *G. hirsutum* AD1 [44]. We further investigated the transfer of *ycf2* fragment by PCR with the following primer pairs: L2-sense (ACCAAAGATGAGCATAGCG); L2-antisense (GAATGGGACGAGAATACCGAA); ycf2-sense (CGTTTGTCTTTACCGCATTC); ycf2-antisense (CCCTACTGAGAGGTCCACTA). The primer L2 was designed to target the nuclear genome flanking the *ycf2* insertion while primer ycf2 were used to verify the *ycf2* gene.

### Phylogenetic analyses and dating of *Gossypium*

The polymorphic SSRs were concatenated to construct a phylogenetic tree with the maximum-likelihood (ML) method. Likelihood analysis was performed in PhyML 3.0 [56] using the HKY model. Support values for nodes on the ML tree were estimated with 100 bootstrap replicates.

For validation of the phylogenetic relationships of *Gossypium* allotetraploids, phylogenetic analyses were performed under maximum-likelihood and Bayesian method using the following data respectively: variable protein-coding genes sequences, intron sequences and variable intergenic sequences. We also constructed a phylogeny of *Gossypium* using the variable sequences. To choose an appropriate outgroup for the phylogenetic analysis, the complete chloroplast genome sequence of *Gossypium hirsutum* was queried using BLAST against the publicly available databases. It showed the highest similarity to the whole-genome sequence of the chloroplast *Theobroma cacao*, another member of Malvaceae [57]. Thus *Theobroma cacao* was chosen as the outgroup for our analysis. Bayesian phylogenetic analyses were performed using MrBayes program [58]. The best-fitting model of nucleotide substitution was determined using both the Akaike information criterion (AIC) and the hierarchical likelihood ratio tests (hLRT) implemented in the program Modeltest 3.7 [59]. Both the AIC and hLRT suggested that the GTR + inv + gamma model was the most appropriate for our data. The Markov chain Monte Carlo (MCMC) algorithm ran for 200,000 generations and the trees sampled each 10 generations for data partition. The first 25% of trees were discarded and the remaining trees were used to construct majority-rule consensus tree for inferring Bayesian posterior probabilities of nodal supports. In all analyses, *Theobroma cacao* was set as outgroup. The Bayesian method was performed with the above-mentioned model. And maximum-likelihood (ML) trees were constructed in MEGA5 program [60] using a General Time Reversible (GTR) model and a rate of Gamma distributed with invariant site (G+I). Sixty-one concatenated protein-coding genes from the 13 taxa (Table S11), including 1 *Ranunculaceae* species [47], 3 *Brassicaceae* species [61], and 2 diploid *Gossypium* species representing dicotyledons; 1 *Acoraceae* species [62], 1 *Typhaceae* species [63], and 2 *Poaceae* species [64], [65], representing monocotyledons; and 2 species of basal angiosperms [47], [66] and 1 gymnosperm [67] forming outgroup, were aligned one by one. The 61 common genes and the analyses of multiple sequences followed previous methods [27], [32]. The divergence time of *Gossypium*, represented by *G. herbaceum* var. *africanum* A1 and *G. raimondii* D5 due to their relative divergent relationships, was calculated. Based on the protein-coding sequences, the values of Ks and Ka were calculated with PAML 4 [68].

To evaluate the constant molecular clock model, 78 concatenated protein-coding sequences were tested for the maximum likelihood-based phylogeny under the General Time Reversible model using likelihood ratio test method implemented in the MEGA5 package [60].

### The polarization of indels

Indels were polarized into insertions and deletions using *Theobroma cacao* as an outgroup. The indels that occurred on the D genome after the split of A and D could be divided into two stages. If 2 A genomes and 9 allotetraploids shared sequence (gap) with the outgroup, then the gap (sequence) the 2 D genomes shared was considered as a deletion (insertion) within D genomes after the split of A genome and D genome and before the split of 2 D genomes, which was defined as the first stage of D-genome divergence. If only one of D genomes had the gap (sequence), then the deletion (insertion) happened after the split of two D genomes, which was defined as the second stage of D-genome divergence.

The indels that arose on the A genome after the divergence of A and D could be divided into three stages. If 2 D genomes had the same sequence (gap) as the outgroup, and the 2 A genomes and 9 allotetraploid genomes shared gap (sequence), then the shared gap (sequence) was considered as a deletion (insertion) in A genome after the divergence of A and D and before the formation of allotetraploids, which was defined as the first stage of A-genome divergence. Conversely, If 2 D genomes and 9 allotetraploid genomes shared sequence with the outgroup, then the shared gap (sequence) by 2 A genomes was considered as a deletion (insertion) in A genomes after the formation of allotetraploid and before the split of 2 A genomes, which was defined as the second stage of A-genome divergence. If only one of A genome existed the gap (sequence), then the deletion (insertion) happened after the split of two A genomes, which was defined as the third stage of A-genome divergence.

The indels in allotetraploids could be divided into two stages. If D genomes and A genomes shared sequence (gap) with the outgroup, then the shared gap (sequence) by allotetraploid genomes was considered as a deletion (insertion) at the first stage. If A genomes, D genomes and partial allotetraploids shared sequence (gap) with outgroup, then the gap (sequence) in other allotetraploids was considered as a deletion (insertion) at the second stage.

## Supporting Information

Figure S1 Phylogenetic tree of 13 *Gossypium* species based on polymorphic SSRs. The polymorphic SSR loci were used to construct phylogenetic trees using maximum-likelihood method.
(TIFF)

Figure S2 The topologies of *Gossypium* allotetraploids. The 3 groups of sequences were used under 2 independent models (Maximum-likelihood model and Bayesian model), respectively. A: 39 variable protein-coding genes using maximum-likelihood method; B: 16 intron sequences using maximum-likelihood method; C: 92 variable intergenic sequences using maximum-likelihood method; D: 39 variable Protein coding genes using Bayesian method; E: 16 intron sequences using Bayesian method; F: 92 variable intergenic sequences using Bayesian method. Numbers above nodes were maximum likelihood bootstrap under Maximum-likelihood model or Bayesian inference posterior probability under Bayesian model.
(TIFF)

Figure S3 Phylogeny of 13 taxa. The common genes of 13 taxa were used to reconstruct the phylogeny. The number on each branch was the nucleotide substitutions per 100 sites.
(TIFF)

Table S1 List of conserved and variable genes in *Gossypium* chloroplast genomes.
(DOC)

Table S2 The AT content (%) of different partitions in 13 chloroplast genomes.
(DOC)

Table S3 The Si/Sv values and total length of indels between genomes.
(DOC)

Table S4 The substitutions and indel number in LSC, SSC and IR regions among 13 *Gossypium* chloroplast genomes.
(DOC)

Table S5 The number of polymorphic SSRs between any two of 13 *Gossypium* chloroplast genomes.
(DOC)

Table S6 The substitution number of protein-coding genes between any two of 13 *Gossypium* chloroplast genomes.
(DOC)

Table S7 Substitutions and indels of intergenic spacers between any two of 13 *Gossypium* chloroplast genomes.
(DOC)

Table S8 The number of indels in different length of chloroplast genomes.
(DOC)

Table S9 The estimated indel ratio between *Gossypium* chloroplast genomes.
(DOC)

Table S10 Primers employed for sequencing.
(DOC)

Table S11 Chloroplast genomes of species adopted in current study.
(DOC)
