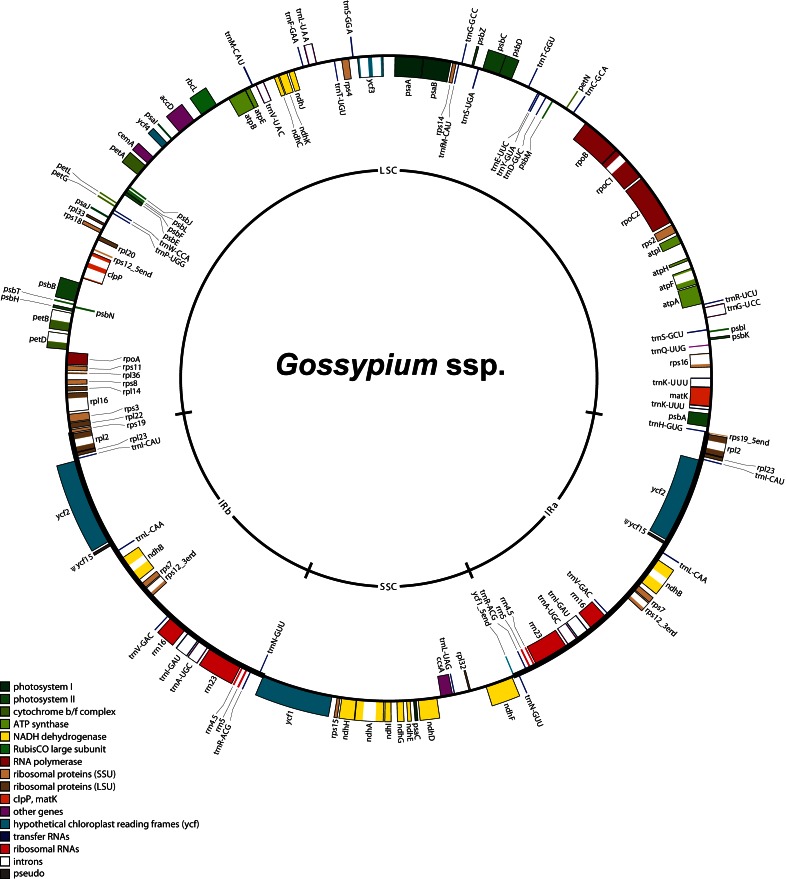# Correction: Analysis of Complete Nucleotide Sequences of 12 *Gossypium* Chloroplast Genomes: Origin and Evolution of Allotetraploids

**DOI:** 10.1371/annotation/47563c17-536c-465d-9b93-cd35a78f6e66

**Published:** 2013-05-17

**Authors:** Qin Xu, Guanjun Xiong, Pengbo Li, Fei He, Yi Huang, Kunbo Wang, Zhaohu Li, Jinping Hua

Figure 1 is cut off at the bottom of the image, so that 6 gene markers are missing. Please see the corrected Figure 1 here: 

**Figure:**