# Supplementary figures and images for: Analysis of Complete Nucleotide Sequences of 12 Gossypium Chloroplast Genomes: Origin and Evolution of Allotetraploids
Source: PLoS One. 2012 Aug 2;7(8):e37128. doi: 10.1371/journal.pone.0037128 (PMC3411646; doi:10.1371/journal.pone.0037128)

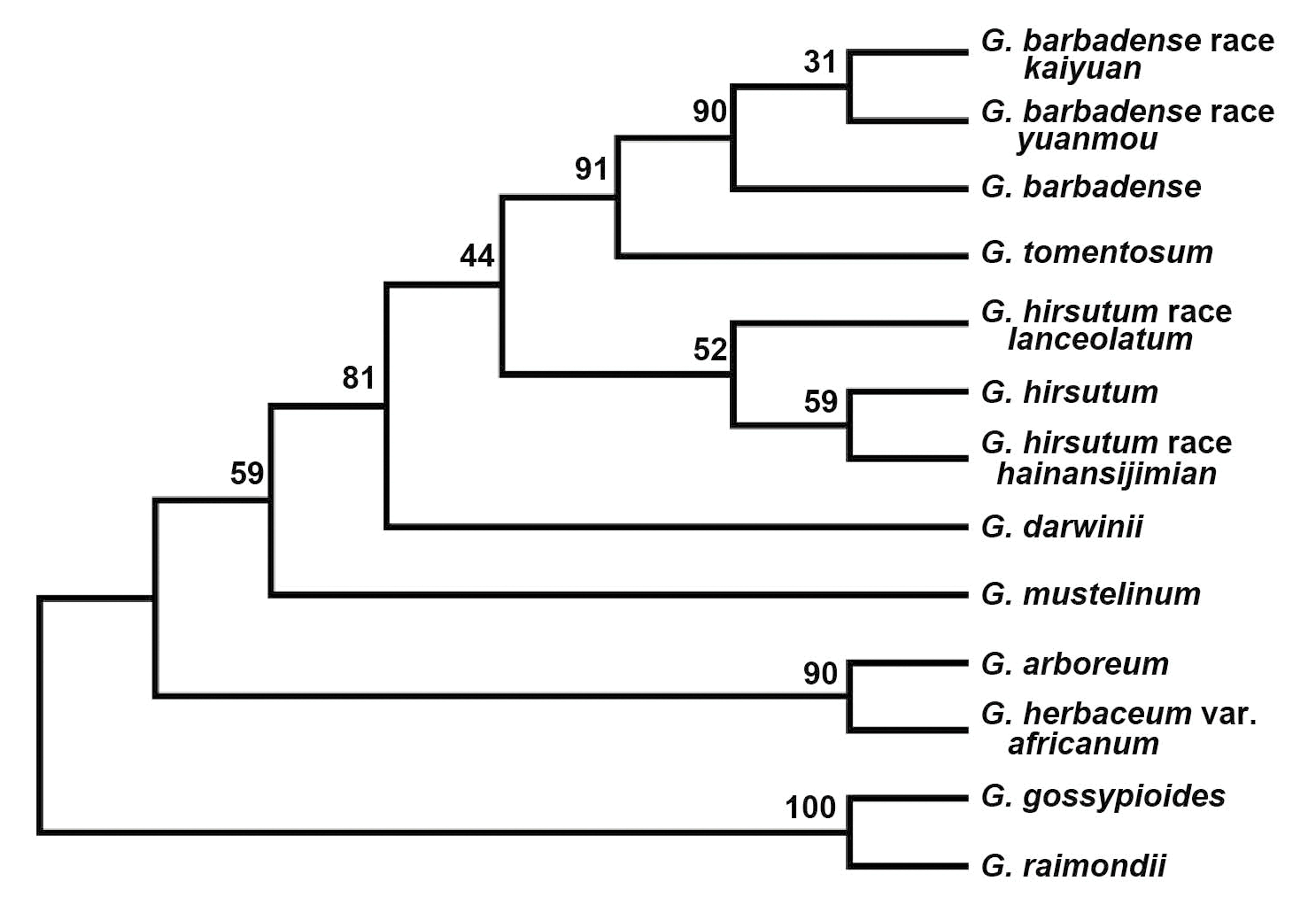

Supplement: Figure S1 — Phylogenetic tree of 13 Gossypium species based on polymorphic SSRs. The polymorphic SSR loci were used to construct phylogenetic trees using maximum-likelihood method. (TIFF) [file pone.0037128.s001.tif]

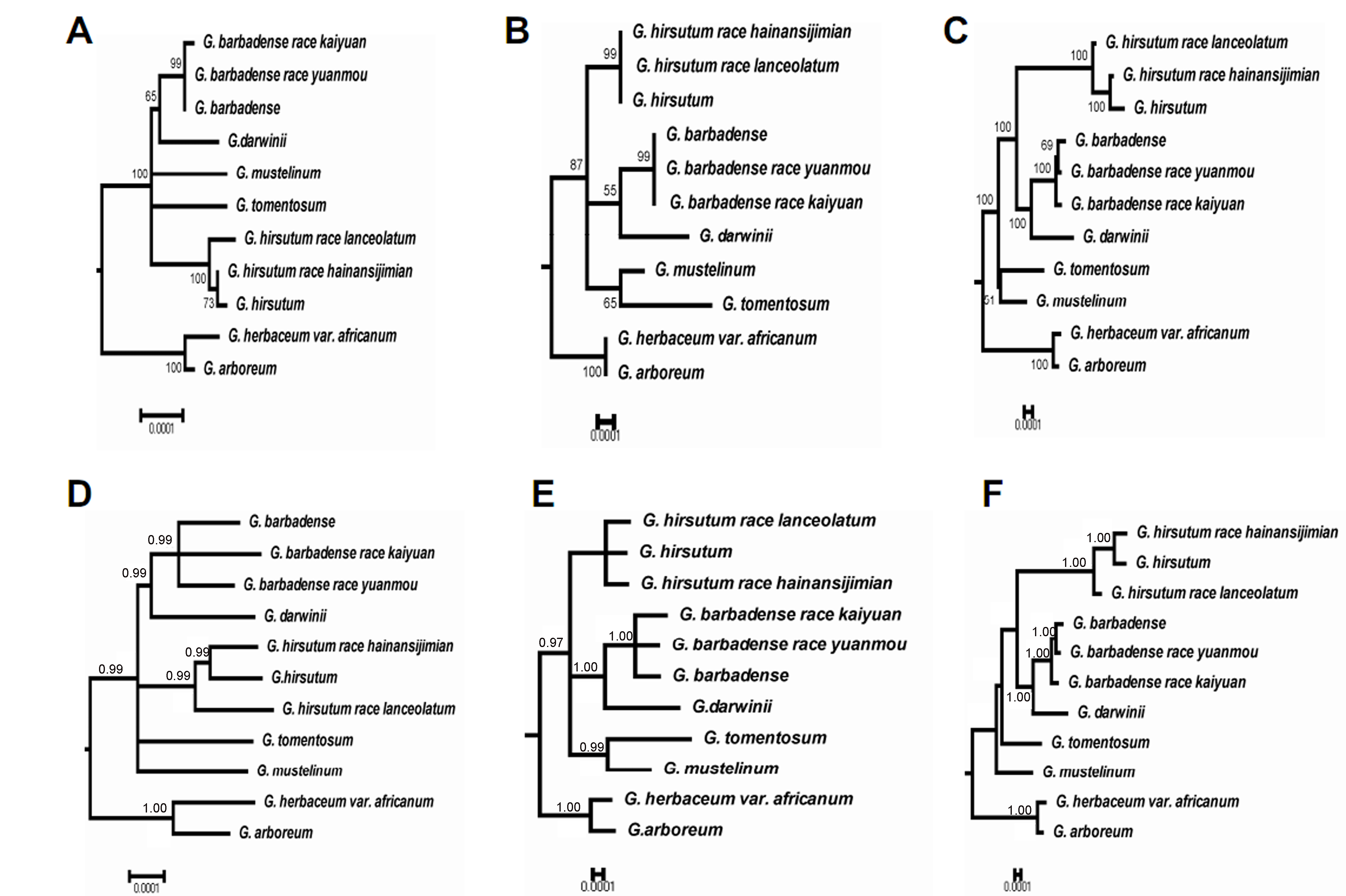

Supplement: Figure S2 — The topologies of Gossypium allotetraploids. The 3 groups of sequences were used under 2 independent models (Maximum-likelihood model and Bayesian model), respectively. A: 39 variable protein-coding genes using maximum-likelihood method; B: 16 intron sequences using maximum-likelihood method; C: 92 variable intergenic sequences using maximum-likelihood method; D: 39 variable Protein coding genes using Bayesian method; E: 16 intron sequences using Bayesian method; F: 92 variable intergenic sequences using Bayesian method. Numbers above nodes were maximum likelihood bootstrap under Maximum-likelihood model or Bayesian inference posterior probability under Bayesian model. (TIFF) [file pone.0037128.s002.tif]

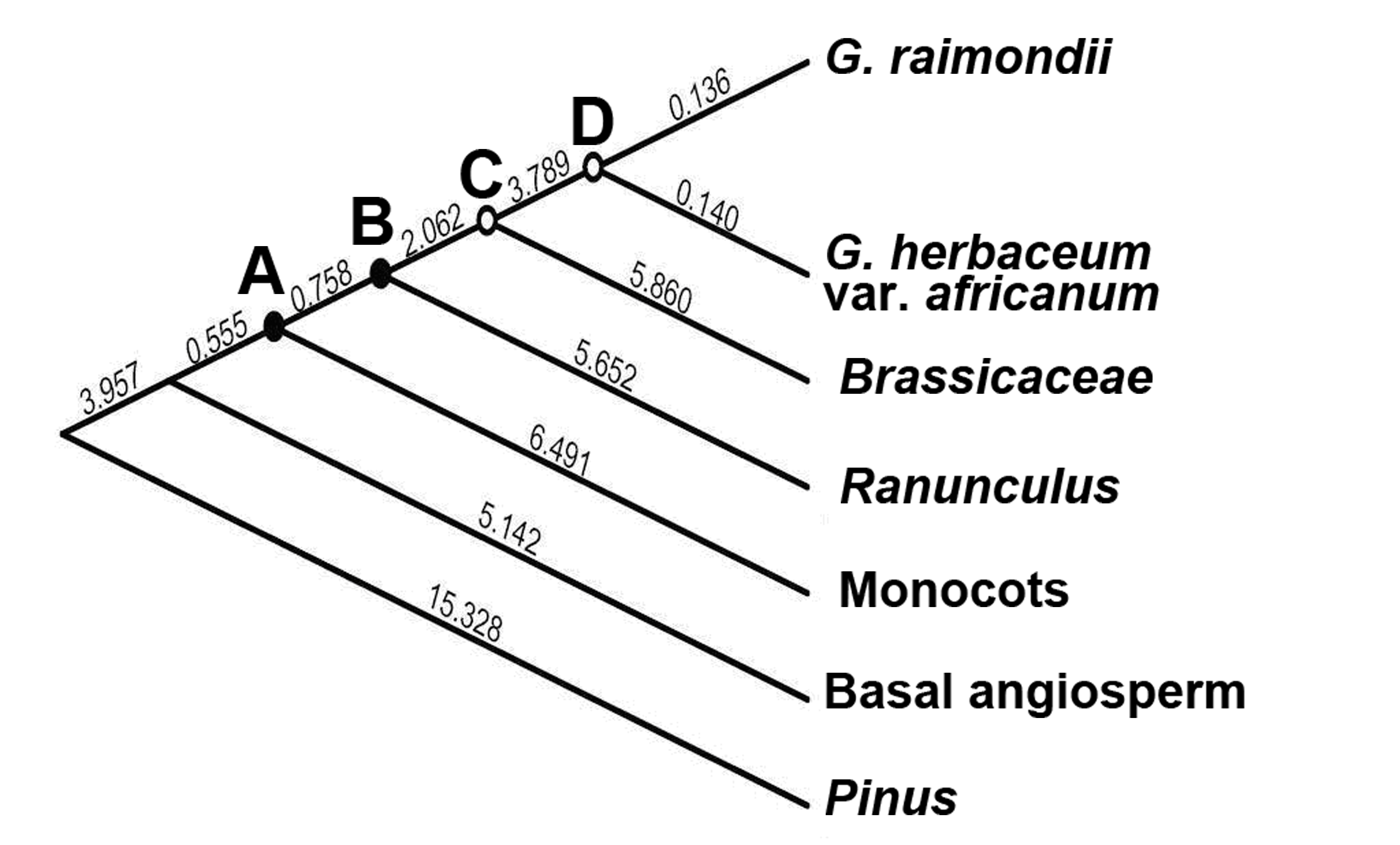

Supplement: Figure S3 — Phylogeny of 13 taxa. The common genes of 13 taxa were used to reconstruct the phylogeny. The number on each branch was the nucleotide substitutions per 100 sites. (TIFF) [file pone.0037128.s003.tif]
