# Supplementary material for: Analysis of Complete Nucleotide Sequences of 12 Gossypium Chloroplast Genomes: Origin and Evolution of Allotetraploids
Source: PLoS One. 2012 Aug 2;7(8):e37128. doi: 10.1371/journal.pone.0037128 (PMC3411646; doi:10.1371/journal.pone.0037128)
Supplement: Table S1 — List of conserved and variable genes in Gossypium chloroplast genomes. (DOC) [file pone.0037128.s004.doc]

**Table S1** List of conserved and variable genes in *Gossypium* chloroplast genomes

| **Gene categories** | **Conserved gene** | **Synonymous mutations** | **Amino acid mutations** |
| --- | --- | --- | --- |
| Photosystem I | *psaI* | *psaA*, *psaB*, *psaC*, *psaJ* |  |
| Photosystem II | *psbE*, *psbF*, *psbH, psbL, psbN, psbT, psbZ* | *psbA*, *psbC*, *psbI, psbJ* | *psbB*, *psbD, psbK, psbM* |
| RuBisCO large subunit |  |  | *rbcL* |
| Cytochrome b/f complex | *petG*, *petL* | *petD*, *petN* | *petA*, *petB* |
| c-type cytochrome |  |  | *ccsA* |
| ATP synthase | *atpF* | *atpH* | *atpA*, *atpB*, *atpE*, *atpI* |
| NADH dehydrogenase | *ndhC, ndhG* | *ndhE, ndhJ*, *ndhK* | *ndhA*, *ndhB**, *ndhD*, *ndhF*, *ndhH*, *ndhI* |
| Assembly/stability of photosystem I |  | *ycf3*, *ycf4* |  |
| RNA polymerase genes |  |  | *rpoA*, *rpoB*, *rpoC1*, *rpoC2* |
| Ribosomal protein | *rps4*, *rps7**, *rps12*, rps14*, *rps18*, *rpl23** | *rps19*, *rpl20*, *rpl32*, *rpl33* | *rps2*, *rps3*, *rps8*, *rps11, rps15*, *rps16*, *rpl2**, *rpl14*, *rpl16*, *rpl36* |
| Ribosomal RNA | *rrn16**, *rrn5**, *rrn4.5** |  | *rrn23 ** |
| Transfer RNA | *trnA_UGC_**, *trnC_GCA_*, *trnD_GUC_*, *trnE_UUC_*, *trnF_GAA_*, *trnG_GCC_*, *trnG_UCC_*, *trnH_GUG_*, *trnI_CAU_**, *trnI_GAU_**, *trnK_UUU_*, *trnL_CAA_*, *trnL_UAA_**, *trnL_UAG_*, *trnM_CAU_*, *trnN_GUU_**, *trnQ_UUG_*, *trnR_ACG_**, *trnR_UCU_*, *trnS_GCU_*, *trnS_GGA_*, *trnS_UGA_*, *trnT_GGU_*, *trnT_UGU_*, *trnV_GAC_**, *trnW_CCA_*, *trnY_GUA_* |  | *trnM_CAU_*, *trnP_UGG_*, *trnW_CCA_* |
| Acetyl-CoA carboxylase subunit |  |  | *accD* |
| Proteolysis subunit |  |  | *clpP* |
| Carbon metabolism |  |  | *cemA* |
| Maturase |  |  | *matK* |
| Conserved reading frames | *ycf15** |  | *ycf1*, *ycf2** |

Note: *These genes have 2 copies in chloroplast genome.
