# Supplementary material for: Analysis of Complete Nucleotide Sequences of 12 Gossypium Chloroplast Genomes: Origin and Evolution of Allotetraploids
Source: PLoS One. 2012 Aug 2;7(8):e37128. doi: 10.1371/journal.pone.0037128 (PMC3411646; doi:10.1371/journal.pone.0037128)
Supplement: Table S2 — The AT content (%) of different partitions in 13 chloroplast genomes. (DOC) [file pone.0037128.s005.doc]

**Table S2** The AT content (%) of different partitions in 13 chloroplast genomes

| **Species** | **Genome** | **LSC** | **SSC** | **IR** | **Non-coding regions** | **Coding regions** | **Introns** | **1st position** | **2nd position** | **3rd position** | **rRNA genes** | **tRNA genes** |
| --- | --- | --- | --- | --- | --- | --- | --- | --- | --- | --- | --- | --- |
| Gaf A1 | 62.8 | 64.8 | 68.4 | 57.1 | 68.4 | 61.8 | 64.7 | 53.8 | 61.8 | 69.6 | 44.5 | 46.9 |
| Ga A2 | 62.8 | 64.8 | 68.4 | 57.1 | 68.4 | 61.7 | 64.6 | 53.7 | 61.7 | 69.8 | 44.5 | 46.9 |
| Gr D5 | 62.7 | 64.7 | 68.3 | 57.0 | 68.2 | 61.8 | 64.5 | 54.0 | 61.7 | 69.6 | 44.5 | 46.9 |
| Gg D6 | 62.7 | 64.7 | 68.2 | 57.0 | 68.2 | 61.8 | 64.5 | 54.0 | 61.7 | 69.6 | 44.5 | 46.9 |
| Gh AD1 | 62.8 | 64.8 | 68.4 | 57.0 | 68.4 | 61.8 | 64.6 | 53.7 | 61.7 | 69.9 | 44.5 | 46.9 |
| Ghh AD1 | 62.7 | 64.8 | 68.4 | 57.0 | 68.3 | 61.8 | 64.7 | 53.7 | 61.7 | 69.9 | 44.5 | 46.9 |
| Ghl AD1 | 62.8 | 64.8 | 68.3 | 57.0 | 68.4 | 61.8 | 64.5 | 53.7 | 61.7 | 69.9 | 44.5 | 46.9 |
| Gb AD2 | 62.8 | 64.8 | 68.3 | 57.0 | 68.4 | 61.8 | 64.6 | 53.8 | 61.9 | 69.6 | 44.5 | 46.9 |
| Gbk AD2 | 62.8 | 64.8 | 68.3 | 57.0 | 68.4 | 61.8 | 64.6 | 53.8 | 61.9 | 69.6 | 44.5 | 46.9 |
| Gby AD2 | 62.8 | 64.8 | 68.3 | 57.0 | 68.4 | 61.8 | 64.6 | 53.8 | 61.9 | 69.6 | 44.5 | 46.9 |
| Gt AD3 | 62.8 | 64.8 | 68.3 | 57.1 | 68.4 | 61.7 | 64.6 | 53.7 | 61.7 | 69.9 | 44.5 | 46.9 |
| Gm AD4 | 62.8 | 64.8 | 68.3 | 57.1 | 68.4 | 61.8 | 64.6 | 54.0 | 61.7 | 69.6 | 44.5 | 46.9 |
| Gd AD5 | 62.8 | 64.8 | 68.3 | 57.0 | 68.4 | 61.8 | 64.6 | 53.8 | 61.9 | 69.6 | 44.5 | 46.9 |

Note: Gaf, Ga, Gr, Gg, Gh, Ghh, Ghl, Gb, Gby, Gbk, Gt, Gm and Gd indicated *G. herbaceum* var. *africanum*, *G. arboreum*, *G. raimondii*, *G. gossypioides*, *G. hirsutum*, *G. hirsutum* race *hainansijimian*, *G. hirsutum* race *lanceolatum*, *G. barbadense*, *G. barbadense* race *yuanmou*, *G. barbadense* race *kaiyuan*, *G. tomentosum*, *G. mustelinum* and *G. darwinii*, respectively. They were the same hereinafter.
