# Supplementary material for: Analysis of Complete Nucleotide Sequences of 12 Gossypium Chloroplast Genomes: Origin and Evolution of Allotetraploids
Source: PLoS One. 2012 Aug 2;7(8):e37128. doi: 10.1371/journal.pone.0037128 (PMC3411646; doi:10.1371/journal.pone.0037128)
Supplement: Table S3 — The Si/Sv values and total length of indels between genomes. (DOC) [file pone.0037128.s006.doc]

**Table S3** The Si/Sv values and total length of indels between genomes

|  | **Gaf A1** | **Ga A2** | **Gh AD1** | **Ghh AD1** | **Ghl AD1** | **Gb AD2** | **Gbk AD2** | **Gby AD2** | **Gt AD3** | **Gm AD4** | **Gd AD5** | **Gr D5** | **Gg D6** |
| --- | --- | --- | --- | --- | --- | --- | --- | --- | --- | --- | --- | --- | --- |
| Gaf A1 |  | 1.20 | 0.44 | 0.44 | 0.45 | 0.41 | 0.42 | 0.41 | 0.37 | 0.45 | 0.42 | 0.56 | 0.62 |
| Ga A2 | 91 |  | 0.43 | 0.44 | 0.45 | 0.41 | 0.45 | 0.44 | 0.38 | 0.44 | 0.41 | 0.56 | 0.62 |
| Gh AD1 | 654 | 621 |  | 1.08 | 0.46 | 0.31 | 0.33 | 0.32 | 0.33 | 0.39 | 0.29 | 0.55 | 0.59 |
| Ghh AD1 | 711 | 676 | 71 |  | 0.39 | 0.31 | 0.35 | 0.33 | 0.32 | 0.38 | 0.36 | 0.54 | 0.60 |
| Ghl AD1 | 661 | 626 | 89 | 126 |  | 0.32 | 0.35 | 0.34 | 0.31 | 0.38 | 0.34 | 0.54 | 0.60 |
| Gb AD2 | 608 | 569 | 484 | 515 | 465 |  | 0.63 | 0.20 | 0.26 | 0.27 | 0.27 | 0.54 | 0.57 |
| Gbk AD2 | 594 | 555 | 468 | 499 | 449 | 18 |  | 0.80 | 0.29 | 0.29 | 0.32 | 0.55 | 0.59 |
| Gby AD2 | 597 | 558 | 467 | 502 | 450 | 17 | 5 |  | 0.26 | 0.26 | 0.29 | 0.55 | 0.59 |
| Gt AD3 | 588 | 501 | 502 | 559 | 509 | 314 | 300 | 303 |  | 0.29 | 0.24 | 0.56 | 0.62 |
| Gm AD4 | 492 | 457 | 496 | 549 | 503 | 376 | 360 | 363 | 338 |  | 0.36 | 0.56 | 0.61 |
| Gd AD5 | 623 | 534 | 415 | 468 | 428 | 211 | 195 | 194 | 255 | 395 |  | 0.55 | 0.59 |
| Gr D5 | 1,512 | 1,427 | 1,586 | 1,617 | 1,601 | 1,632 | 1,618 | 1,621 | 1,626 | 1,550 | 1,653 |  | 0.55 |
| Gg D6 | 1,738 | 1,651 | 1,796 | 1,801 | 1,783 | 1,814 | 1,800 | 1,803 | 1,826 | 1,772 | 1,863 | 486 |  |

Note: The upper triangle showed the Si/Sv values and the lower triangle showed the total length of indels.
