# Supplementary material for: Analysis of Complete Nucleotide Sequences of 12 Gossypium Chloroplast Genomes: Origin and Evolution of Allotetraploids
Source: PLoS One. 2012 Aug 2;7(8):e37128. doi: 10.1371/journal.pone.0037128 (PMC3411646; doi:10.1371/journal.pone.0037128)
Supplement: Table S4 — The substitutions and indel number in LSC, SSC and IR regions among 13 Gossypium chloroplast genomes. (DOC) [file pone.0037128.s007.doc]

**Table S4** The substitutions and indel number in LSC, SSC and IR regions among 13 *Gossypium* chloroplast genomes

|  | **DNA substitutions** | | | **Indel length** | | | **Indel NO.** | | |
| --- | --- | --- | --- | --- | --- | --- | --- | --- | --- |
| **Genome** | **LSC** | **IR** | **SSC** | **LSC** | **IR** | **SSC** | **LSC** | **IR** | **SSC** |
| Ga-Gaf | 6 | 1 | 4 | 77 | 3 | 11 | 10 | 1 | 1 |
| Ga-Gr | 605 | 24 | 201 | 1208 | 101 | 157 | 212 | 9 | 29 |
| Ga-Gg | 662 | 24 | 210 | 1173 | 53 | 371 | 211 | 11 | 29 |
| Gaf-Gg | 665 | 25 | 210 | 1243 | 56 | 382 | 216 | 11 | 30 |
| Gaf-Gr | 608 | 25 | 201 | 1276 | 104 | 168 | 217 | 9 | 30 |
| Gg-Gr | 120 | 6 | 53 | 209 | 74 | 206 | 57 | 3 | 9 |
| Ga-Gd | 115 | 7 | 42 | 436 | 26 | 46 | 82 | 5 | 12 |
| Ga-Gh | 156 | 7 | 51 | 503 | 35 | 48 | 91 | 7 | 14 |
| Ga-Gm | 100 | 4 | 39 | 364 | 24 | 49 | 86 | 6 | 12 |
| Ga-Gbk | 114 | 7 | 36 | 426 | 36 | 45 | 85 | 7 | 12 |
| Ga-Gby | 112 | 6 | 37 | 430 | 36 | 45 | 86 | 7 | 12 |
| Ga-Ghl | 151 | 9 | 46 | 513 | 45 | 39 | 88 | 9 | 11 |
| Ga-Ghh | 168 | 7 | 45 | 548 | 35 | 47 | 101 | 7 | 14 |
| Ga-Gt | 112 | 6 | 36 | 386 | 38 | 39 | 85 | 7 | 10 |
| Ga-Gb | 113 | 7 | 38 | 441 | 36 | 45 | 86 | 7 | 12 |
| Gaf-Gd | 118 | 8 | 44 | 508 | 29 | 57 | 88 | 6 | 13 |
| Gaf-Gh | 157 | 8 | 53 | 519 | 38 | 59 | 92 | 8 | 15 |
| Gaf-Ghl | 152 | 10 | 48 | 531 | 48 | 50 | 90 | 9 | 12 |
| Gaf-Gm | 101 | 5 | 41 | 382 | 27 | 60 | 88 | 6 | 13 |
| Gaf-Ghh | 169 | 8 | 47 | 566 | 38 | 58 | 103 | 8 | 15 |
| Gaf-Gt | 110 | 7 | 38 | 456 | 41 | 50 | 90 | 8 | 11 |
| Gaf-Gb | 111 | 8 | 40 | 463 | 39 | 56 | 91 | 8 | 13 |
| Gaf-Gbk | 112 | 8 | 38 | 448 | 39 | 56 | 90 | 8 | 13 |
| Gaf-Gby | 110 | 7 | 39 | 452 | 39 | 56 | 91 | 8 | 13 |
| Ghl-Gr | 689 | 30 | 192 | 1345 | 124 | 162 | 209 | 12 | 27 |
| Gm-Gr | 618 | 24 | 193 | 1304 | 109 | 174 | 213 | 11 | 29 |
| Ghh-Gr | 682 | 30 | 190 | 1344 | 116 | 168 | 217 | 12 | 27 |
| Gd-Gr | 622 | 27 | 196 | 1390 | 117 | 167 | 218 | 12 | 29 |
| Gh-Gr | 683 | 30 | 196 | 1323 | 116 | 169 | 213 | 12 | 27 |
| Gr-Gbk | 628 | 31 | 190 | 1342 | 117 | 168 | 219 | 13 | 29 |
| Gr-Gby | 626 | 30 | 191 | 1346 | 117 | 168 | 220 | 13 | 29 |
| Gr-Gt | 620 | 26 | 190 | 1346 | 129 | 162 | 217 | 14 | 27 |
| Gb-Gr | 628 | 31 | 192 | 1357 | 117 | 168 | 221 | 13 | 29 |
| Gd-Gg | 667 | 27 | 190 | 1351 | 69 | 371 | 222 | 14 | 28 |
| Gg-Gh | 737 | 30 | 193 | 1284 | 68 | 373 | 215 | 14 | 26 |
| Gg-Gm | 676 | 24 | 196 | 1267 | 65 | 378 | 214 | 13 | 28 |
| Gg-Gbk | 670 | 31 | 193 | 1295 | 69 | 369 | 219 | 15 | 29 |
| Gg-Gby | 671 | 30 | 197 | 1301 | 69 | 372 | 223 | 15 | 28 |
| Gg-Ghl | 746 | 31 | 199 | 1286 | 70 | 366 | 207 | 14 | 26 |
| Gg-Ghh | 731 | 30 | 197 | 1301 | 68 | 372 | 218 | 14 | 26 |
| Gg-Gt | 686 | 26 | 195 | 1297 | 81 | 366 | 218 | 16 | 26 |
| Gb-Gg | 673 | 31 | 197 | 1312 | 69 | 372 | 224 | 15 | 28 |
| Gd-Gh | 119 | 6 | 20 | 363 | 19 | 14 | 69 | 4 | 5 |
| Gd-Gbk | 48 | 4 | 10 | 162 | 10 | 1 | 27 | 2 | 1 |
| Gd-Gby | 46 | 3 | 11 | 162 | 10 | 1 | 27 | 2 | 1 |
| Gd-Ghl | 122 | 8 | 22 | 377 | 29 | 9 | 66 | 6 | 4 |
| Gh-Ghl | 17 | 4 | 10 | 78 | 10 | 7 | 18 | 2 | 2 |
| Ghl-Gm | 137 | 5 | 20 | 449 | 25 | 24 | 70 | 4 | 8 |
| Ghl-Gbk | 113 | 8 | 16 | 409 | 29 | 8 | 67 | 6 | 3 |
| Ghl-Gby | 111 | 7 | 17 | 411 | 29 | 8 | 68 | 6 | 3 |
| Gd-Gm | 88 | 3 | 16 | 354 | 8 | 29 | 66 | 3 | 6 |
| Gh-Gm | 132 | 3 | 26 | 437 | 17 | 29 | 74 | 5 | 9 |
| Gm-Gbk | 73 | 3 | 10 | 286 | 18 | 30 | 68 | 5 | 7 |
| Gm-Gby | 71 | 2 | 11 | 290 | 18 | 30 | 70 | 5 | 7 |
| Gd-Ghh | 132 | 6 | 24 | 406 | 19 | 13 | 79 | 4 | 4 |
| Gh-Ghh | 19 | 0 | 6 | 59 | 0 | 1 | 16 | 0 | 1 |
| Ghl-Ghh | 31 | 4 | 4 | 127 | 10 | 6 | 29 | 2 | 2 |
| Gm-Ghh | 147 | 3 | 20 | 480 | 17 | 28 | 79 | 5 | 8 |
| Ghh-Gbk | 123 | 6 | 18 | 448 | 19 | 12 | 76 | 4 | 3 |
| Ghh-Gby | 127 | 5 | 19 | 452 | 19 | 12 | 81 | 4 | 3 |
| Gd-Gt | 81 | 5 | 13 | 222 | 12 | 9 | 40 | 2 | 4 |
| Gh-Gt | 133 | 5 | 23 | 429 | 31 | 11 | 72 | 6 | 6 |
| Ghl-Gt | 132 | 7 | 17 | 439 | 41 | 4 | 67 | 8 | 4 |
| Gm-Gt | 71 | 2 | 9 | 278 | 20 | 24 | 66 | 5 | 6 |
| Ghh-Gt | 147 | 5 | 17 | 476 | 31 | 10 | 81 | 6 | 6 |
| Gt-Gbk | 72 | 3 | 7 | 234 | 22 | 10 | 47 | 4 | 5 |
| Gt-Gby | 70 | 2 | 8 | 238 | 22 | 10 | 49 | 4 | 5 |
| Gb-Gd | 47 | 4 | 11 | 179 | 10 | 1 | 29 | 2 | 1 |
| Gb-Gh | 115 | 6 | 25 | 422 | 19 | 13 | 71 | 4 | 4 |
| Gb-Ghl | 112 | 8 | 18 | 426 | 29 | 8 | 69 | 6 | 3 |
| Gb-Gm | 71 | 3 | 12 | 303 | 18 | 30 | 71 | 5 | 7 |
| Gb-Ghh | 128 | 6 | 20 | 465 | 19 | 12 | 82 | 4 | 3 |
| Gb-Gt | 71 | 3 | 9 | 249 | 22 | 10 | 49 | 4 | 5 |
| Gb-Gbk | 7 | 2 | 2 | 17 | 0 | 0 | 4 | 0 | 0 |
| Gb-Gby | 1 | 1 | 3 | 17 | 0 | 0 | 3 | 0 | 0 |
| Gh-Gbk | 116 | 6 | 24 | 405 | 19 | 13 | 69 | 4 | 4 |
| Gh-Gby | 114 | 5 | 25 | 405 | 19 | 13 | 69 | 4 | 4 |
| Gbk-Gby | 6 | 1 | 1 | 4 | 0 | 0 | 4 | 0 | 0 |
