# Supplementary material for: Analysis of Complete Nucleotide Sequences of 12 Gossypium Chloroplast Genomes: Origin and Evolution of Allotetraploids
Source: PLoS One. 2012 Aug 2;7(8):e37128. doi: 10.1371/journal.pone.0037128 (PMC3411646; doi:10.1371/journal.pone.0037128)
Supplement: Table S5 — The number of polymorphic SSRs between any two of 13 Gossypium chloroplast genomes. (DOC) [file pone.0037128.s008.doc]

**Table S5** The number of polymorphic SSRs between any two of 13 *Gossypium* chloroplast genomes

|  | **Gaf A1** | **Ga A2** | **Gh AD1** | **Ghh AD1** | **Ghl AD1** | **Gb AD2** | **Gbk AD2** | **Gby AD2** | **Gt AD3** | **Gm AD4** | **Gd AD5** | **Gr D5** |
| --- | --- | --- | --- | --- | --- | --- | --- | --- | --- | --- | --- | --- |
| Gaf A1 |  |  |  |  |  |  |  |  |  |  |  |  |
| Ga A2 | 7 |  |  |  |  |  |  |  |  |  |  |  |
| Gh AD1 | 43 | 40 |  |  |  |  |  |  |  |  |  |  |
| Ghh AD1 | 44 | 40 | 7 |  |  |  |  |  |  |  |  |  |
| Ghl AD1 | 38 | 36 | 17 | 17 |  |  |  |  |  |  |  |  |
| Gb AD2 | 45 | 40 | 30 | 32 | 32 |  |  |  |  |  |  |  |
| Gbk AD2 | 42 | 37 | 32 | 32 | 35 | 3 |  |  |  |  |  |  |
| Gby AD2 | 43 | 38 | 33 | 33 | 32 | 3 | 2 |  |  |  |  |  |
| Gt AD3 | 45 | 42 | 37 | 38 | 31 | 25 | 21 | 22 |  |  |  |  |
| Gm AD4 | 42 | 39 | 36 | 34 | 30 | 36 | 32 | 34 | 33 |  |  |  |
| Gd AD5 | 42 | 35 | 27 | 30 | 30 | 18 | 16 | 16 | 22 | 27 |  |  |
| Gr D5 | 117 | 113 | 117 | 117 | 115 | 124 | 123 | 124 | 124 | 115 | 120 |  |
| Gg D6 | 119 | 115 | 117 | 118 | 115 | 128 | 127 | 127 | 128 | 117 | 121 | 40 |
