# Supplementary material for: Analysis of Complete Nucleotide Sequences of 12 Gossypium Chloroplast Genomes: Origin and Evolution of Allotetraploids
Source: PLoS One. 2012 Aug 2;7(8):e37128. doi: 10.1371/journal.pone.0037128 (PMC3411646; doi:10.1371/journal.pone.0037128)
Supplement: Table S6 — The substitution number of protein-coding genes between any two of 13 Gossypium chloroplast genomes. (DOC) [file pone.0037128.s009.doc]

**Table S6** The substitution number of protein-coding genes between any two of 13 *Gossypium* chloroplast genomes

|  | **Ghh** | **Gh** | **Ghl** | **Gb** | **Gbk** | **Gby** | **Gd** | **Gt** | **Gm** | **Gaf** | **Ga** | **Gg** | **Gr** |
| --- | --- | --- | --- | --- | --- | --- | --- | --- | --- | --- | --- | --- | --- |
| Ghh |  | 0 | 3 | 7 | 7 | 7 | 10 | 7 | 10 | 15 | 13 | 117 | 119 |
| Gh | 1 |  | 3 | 7 | 7 | 7 | 10 | 7 | 10 | 15 | 13 | 117 | 119 |
| Ghl | 4 | 5 |  | 8 | 8 | 8 | 11 | 8 | 11 | 16 | 14 | 118 | 120 |
| Gb | 15 | 16 | 17 |  | 0 | 0 | 7 | 6 | 9 | 14 | 12 | 114 | 116 |
| Gbk | 16 | 17 | 18 | 1 |  | 0 | 7 | 6 | 9 | 14 | 12 | 114 | 116 |
| Gby | 15 | 16 | 17 | 0 | 1 |  | 7 | 6 | 9 | 14 | 12 | 114 | 116 |
| Gd | 19 | 20 | 21 | 10 | 11 | 10 |  | 9 | 10 | 17 | 15 | 119 | 121 |
| Gt | 20 | 21 | 22 | 13 | 14 | 13 | 17 |  | 9 | 14 | 12 | 116 | 118 |
| Gm | 20 | 21 | 22 | 13 | 14 | 13 | 16 | 18 |  | 17 | 15 | 118 | 120 |
| Gaf | 36 | 37 | 38 | 29 | 30 | 29 | 33 | 34 | 34 |  | 2 | 110 | 114 |
| Ga | 32 | 33 | 34 | 25 | 26 | 25 | 29 | 30 | 30 | 6 |  | 108 | 112 |
| Gg | 255 | 256 | 257 | 241 | 242 | 241 | 252 | 253 | 252 | 251 | 249 |  | 34 |
| Gr | 257 | 258 | 259 | 243 | 244 | 243 | 254 | 255 | 254 | 255 | 253 | 60 |  |

Note: The substitutions of protein-coding genes at the DNA level were in the lower triangle and those at the amino acid level were in the upper triangle.
