# Supplementary material for: Analysis of Complete Nucleotide Sequences of 12 Gossypium Chloroplast Genomes: Origin and Evolution of Allotetraploids
Source: PLoS One. 2012 Aug 2;7(8):e37128. doi: 10.1371/journal.pone.0037128 (PMC3411646; doi:10.1371/journal.pone.0037128)
Supplement: Table S7 — Substitutions and indels of intergenic spacers between any two of 13 Gossypium chloroplast genomes. (DOC) [file pone.0037128.s010.doc]

**Table S7** Substitutions and indels of intergenic spacers between any two of 13 *Gossypium* chloroplast genomes

**Table S7A** Substitutions and Si/Sv values of intergenic spacers

|  | **Gaf A1** | **Ga A2** | **Gh AD1** | **Ghh AD1** | **Ghl AD1** | **Gb AD2** | **Gbk AD2** | **Gby AD2** | **Gt AD3** | **Gm AD4** | **Gd AD5** | **Gr D5** | **Gg D6** |
| --- | --- | --- | --- | --- | --- | --- | --- | --- | --- | --- | --- | --- | --- |
| Gaf A1 |  | 0.75 | 0.34 | 0.34 | 0.34 | 0.32 | 0.35 | 0.32 | 0.27 | 0.34 | 0.34 | 0.44 | 0.54 |
| Ga A2 | 7 |  | 0.33 | 0.34 | 0.34 | 0.32 | 0.35 | 0.32 | 0.27 | 0.34 | 0.34 | 0.44 | 0.53 |
| Gh AD1 | 179 | 176 |  | 1.09 | 0.30 | 0.24 | 0.28 | 0.26 | 0.24 | 0.33 | 0.21 | 0.41 | 0.47 |
| Ghh AD1 | 185 | 182 | 23 |  | 0.31 | 0.26 | 0.30 | 0.26 | 0.26 | 0.31 | 0.30 | 0.41 | 0.49 |
| Ghl AD1 | 170 | 167 | 26 | 34 |  | 0.23 | 0.25 | 0.22 | 0.21 | 0.27 | 0.26 | 0.41 | 0.49 |
| Gb AD2 | 127 | 124 | 122 | 130 | 114 |  | 1.25 | 0.25 | 0.20 | 0.20 | 0.30 | 0.42 | 0.48 |
| Gbk AD2 | 127 | 124 | 123 | 131 | 111 | 9 |  | 2.00 | 0.25 | 0.25 | 0.42 | 0.42 | 0.48 |
| Gby AD2 | 127 | 124 | 123 | 130 | 111 | 5 | 6 |  | 0.20 | 0.21 | 0.33 | 0.41 | 0.47 |
| Gt AD3 | 117 | 114 | 130 | 139 | 126 | 60 | 60 | 60 |  | 0.26 | 0.22 | 0.43 | 0.53 |
| Gm AD4 | 114 | 111 | 134 | 143 | 128 | 65 | 64 | 64 | 58 |  | 0.34 | 0.42 | 0.51 |
| Gd AD5 | 129 | 126 | 114 | 131 | 118 | 43 | 44 | 44 | 67 | 79 |  | 0.43 | 0.49 |
| Gr D5 | 517 | 513 | 585 | 578 | 585 | 540 | 535 | 534 | 512 | 516 | 520 |  | 0.63 |
| Gg D6 | 607 | 602 | 663 | 661 | 673 | 614 | 611 | 609 | 609 | 611 | 592 | 127 |  |

Note: The upper triangle showed the Si/Sv values and the lower triangle showed the total substitutions.

**Table S7B** Number and total length of indels for intergenic spacers

|  | **Gaf A1** | **Ga A2** | **Gh AD1** | **Ghh AD1** | **Ghl AD1** | **Gb AD2** | **Gbk AD2** | **Gby AD2** | **Gt AD3** | **Gm AD4** | **Gd AD5** | **Gr D5** | **Gg D6** |
| --- | --- | --- | --- | --- | --- | --- | --- | --- | --- | --- | --- | --- | --- |
| Gaf A1 |  | 11 | 100 | 109 | 97 | 94 | 96 | 96 | 96 | 91 | 88 | 216 | 224 |
| Ga A2 | 67 |  | 96 | 104 | 94 | 88 | 90 | 90 | 90 | 88 | 80 | 211 | 218 |
| Gh AD1 | 547 | 536 |  | 13 | 19 | 69 | 70 | 70 | 79 | 79 | 68 | 215 | 223 |
| Ghh AD1 | 594 | 581 | 55 |  | 29 | 77 | 77 | 78 | 86 | 82 | 74 | 217 | 225 |
| Ghl AD1 | 552 | 539 | 81 | 130 |  | 69 | 69 | 69 | 75 | 71 | 66 | 210 | 217 |
| Gb AD2 | 450 | 435 | 413 | 454 | 414 |  | 6 | 3 | 52 | 66 | 24 | 219 | 229 |
| Gbk AD2 | 454 | 439 | 415 | 456 | 402 | 16 |  | 4 | 53 | 66 | 25 | 219 | 229 |
| Gby AD2 | 456 | 441 | 415 | 458 | 402 | 14 | 4 |  | 53 | 67 | 25 | 219 | 229 |
| Gt AD3 | 475 | 412 | 474 | 519 | 479 | 249 | 253 | 255 |  | 66 | 38 | 221 | 229 |
| Gm AD4 | 397 | 384 | 460 | 503 | 463 | 287 | 297 | 299 | 302 |  | 60 | 212 | 218 |
| Gd AD5 | 493 | 428 | 372 | 411 | 381 | 163 | 165 | 165 | 220 | 344 |  | 216 | 225 |
| Gr D5 | 1244 | 1181 | 1329 | 1350 | 1342 | 1314 | 1324 | 1326 | 1359 | 1281 | 1371 |  | 54 |
| Gg D6 | 1529 | 1464 | 1600 | 1617 | 1609 | 1581 | 1591 | 1593 | 1622 | 1546 | 1642 | 361 |  |

Note: The upper triangle showed the number of indels and the lower triangle showed total length of indels.
