# Supplementary material for: Analysis of Complete Nucleotide Sequences of 12 Gossypium Chloroplast Genomes: Origin and Evolution of Allotetraploids
Source: PLoS One. 2012 Aug 2;7(8):e37128. doi: 10.1371/journal.pone.0037128 (PMC3411646; doi:10.1371/journal.pone.0037128)
Supplement: Table S8 — The number of indels in different length of chloroplast genomes. (DOC) [file pone.0037128.s011.doc]

**Table S8** The number of indels in different length of chloroplast genomes

**Table S8A** The number of indels in different length between any two 13 *Gossypium* plastomes

|  | **1 bp** | **2 bp** | **3 bp** | **4 bp** | **5 bp** | **6 bp** | **7 bp** | **8 bp** | **9 bp** | **10 bp** |
| --- | --- | --- | --- | --- | --- | --- | --- | --- | --- | --- |
| Ga-Gaf | 6 | 0 | 2 | 0 | 0 | 1 | 0 | 0 | 0 | 0 |
| Ga-Gr | 78 | 32 | 9 | 10 | 46 | 20 | 14 | 7 | 4 | 8 |
| Gg-Ga | 74 | 30 | 15 | 7 | 51 | 21 | 14 | 6 | 6 | 6 |
| Gg-Gaf | 75 | 28 | 14 | 8 | 53 | 23 | 13 | 5 | 7 | 6 |
| Gr-Gaf | 81 | 29 | 8 | 9 | 48 | 23 | 13 | 6 | 5 | 8 |
| Gg-Gr | 43 | 7 | 2 | 2 | 5 | 3 | 2 | 0 | 0 | 0 |
| Ga-Gd | 29 | 6 | 3 | 4 | 24 | 23 | 6 | 2 | 1 | 0 |
| Ga-Gh | 33 | 11 | 2 | 7 | 25 | 21 | 4 | 4 | 1 | 1 |
| Ga-Gm | 37 | 8 | 0 | 6 | 21 | 18 | 9 | 3 | 1 | 0 |
| Ga-Gbk | 31 | 9 | 3 | 4 | 28 | 21 | 6 | 2 | 0 | 0 |
| Ga-Gby | 30 | 9 | 4 | 3 | 29 | 21 | 6 | 2 | 0 | 0 |
| Ghl-Ga | 36 | 7 | 1 | 8 | 24 | 19 | 4 | 4 | 1 | 1 |
| Ghh-Ga | 40 | 8 | 3 | 7 | 28 | 21 | 5 | 4 | 1 | 1 |
| Gt-Ga | 35 | 6 | 3 | 4 | 20 | 25 | 6 | 4 | 0 | 0 |
| Gb-Ga | 30 | 8 | 5 | 2 | 29 | 21 | 6 | 2 | 0 | 0 |
| Gd-Gaf | 29 | 7 | 7 | 3 | 24 | 24 | 6 | 2 | 1 | 0 |
| Gh-Gaf | 33 | 10 | 4 | 7 | 25 | 22 | 4 | 4 | 1 | 1 |
| Ghl-Gaf | 34 | 7 | 1 | 10 | 24 | 20 | 4 | 4 | 1 | 1 |
| Gm-Gaf | 36 | 6 | 1 | 8 | 21 | 19 | 9 | 3 | 1 | 0 |
| Ghh-Gaf | 41 | 7 | 4 | 8 | 28 | 22 | 5 | 4 | 1 | 1 |
| Gt-Gaf | 35 | 7 | 5 | 4 | 20 | 26 | 6 | 4 | 0 | 0 |
| Gb-Gaf | 33 | 8 | 7 | 2 | 28 | 23 | 6 | 2 | 0 | 0 |
| Gbk-Gaf | 34 | 8 | 7 | 2 | 29 | 22 | 6 | 2 | 0 | 0 |
| Gby-Gaf | 33 | 8 | 8 | 2 | 28 | 23 | 6 | 2 | 0 | 0 |
| Ghl-Gr | 73 | 30 | 13 | 11 | 45 | 23 | 9 | 8 | 5 | 4 |
| Gm-Gr | 71 | 28 | 14 | 11 | 45 | 26 | 9 | 10 | 4 | 4 |
| Ghh-Gr | 77 | 31 | 16 | 11 | 44 | 26 | 9 | 7 | 5 | 4 |
| Gr-Gd | 73 | 30 | 13 | 11 | 45 | 27 | 13 | 10 | 5 | 6 |
| Gr-Gh | 76 | 30 | 15 | 11 | 45 | 25 | 9 | 7 | 5 | 4 |
| Gr-Gbk | 73 | 36 | 12 | 10 | 51 | 27 | 11 | 6 | 6 | 5 |
| Gr-Gby | 73 | 36 | 11 | 11 | 49 | 29 | 11 | 6 | 6 | 5 |
| Gt-Gr | 69 | 34 | 14 | 9 | 45 | 31 | 11 | 10 | 5 | 4 |
| Gb-Gr | 73 | 36 | 12 | 11 | 49 | 28 | 11 | 6 | 6 | 5 |
| Gg-Gd | 76 | 31 | 14 | 9 | 49 | 28 | 15 | 7 | 6 | 3 |
| Gg-Gh | 77 | 32 | 13 | 11 | 47 | 28 | 8 | 7 | 5 | 3 |
| Gg-Gm | 77 | 26 | 8 | 13 | 47 | 27 | 10 | 8 | 5 | 4 |
| Gg-Gbk | 80 | 30 | 15 | 10 | 53 | 27 | 13 | 6 | 5 | 3 |
| Gg-Gby | 78 | 32 | 15 | 9 | 54 | 27 | 13 | 6 | 5 | 3 |
| Ghl-Gg | 71 | 31 | 12 | 10 | 47 | 26 | 8 | 8 | 5 | 3 |
| Ghh-Gg | 80 | 29 | 17 | 10 | 45 | 27 | 10 | 7 | 5 | 3 |
| Gt-Gg | 74 | 31 | 13 | 9 | 48 | 31 | 11 | 9 | 5 | 3 |
| Gb-Gg | 79 | 30 | 16 | 8 | 54 | 28 | 12 | 6 | 6 | 4 |
| Gd-Gh | 25 | 8 | 2 | 5 | 20 | 12 | 3 | 3 | 1 | 0 |
| Gd-Gbk | 13 | 2 | 2 | 0 | 11 | 2 | 0 | 0 | 0 | 0 |
| Gd-Gby | 12 | 3 | 0 | 1 | 11 | 2 | 0 | 0 | 0 | 0 |
| Ghl-Gd | 29 | 4 | 1 | 5 | 20 | 9 | 3 | 3 | 1 | 0 |
| Ghl-Gh | 10 | 4 | 0 | 0 | 3 | 3 | 0 | 0 | 0 | 0 |
| Ghl-Gm | 30 | 4 | 0 | 5 | 18 | 10 | 5 | 4 | 1 | 0 |
| Ghl-Gbk | 29 | 4 | 3 | 5 | 19 | 9 | 3 | 2 | 1 | 0 |
| Ghl-Gby | 29 | 4 | 2 | 6 | 19 | 9 | 3 | 2 | 1 | 0 |
| Gm-Gd | 23 | 8 | 2 | 3 | 15 | 11 | 4 | 3 | 1 | 0 |
| Gm-Gh | 30 | 7 | 1 | 5 | 19 | 14 | 5 | 4 | 1 | 0 |
| Gm-Gbk | 33 | 5 | 3 | 3 | 16 | 11 | 3 | 3 | 1 | 0 |
| Gm-Gby | 33 | 5 | 4 | 3 | 16 | 11 | 3 | 3 | 1 | 0 |
| Ghh-Gd | 29 | 7 | 2 | 6 | 22 | 13 | 4 | 3 | 1 | 0 |
| Ghh-Gh | 8 | 2 | 1 | 1 | 2 | 1 | 1 | 0 | 0 | 0 |
| Ghh-Ghl | 17 | 2 | 1 | 0 | 5 | 3 | 2 | 0 | 0 | 0 |
| Ghh-Gm | 33 | 5 | 1 | 5 | 19 | 15 | 5 | 3 | 1 | 0 |
| Ghh-Gbk | 31 | 4 | 3 | 5 | 22 | 14 | 4 | 2 | 1 | 0 |
| Ghh-Gby | 30 | 6 | 3 | 5 | 22 | 14 | 4 | 2 | 1 | 0 |
| Gt-Gd | 18 | 2 | 1 | 0 | 9 | 10 | 3 | 1 | 0 | 0 |
| Gt-Gh | 29 | 9 | 0 | 5 | 19 | 16 | 4 | 3 | 0 | 0 |
| Gt-Ghl | 31 | 4 | 0 | 5 | 18 | 13 | 4 | 3 | 0 | 0 |
| Gt-Gm | 32 | 5 | 0 | 3 | 10 | 16 | 7 | 3 | 1 | 0 |
| Gt-Ghh | 34 | 8 | 2 | 4 | 21 | 16 | 5 | 3 | 0 | 0 |
| Gt-Gbk | 20 | 4 | 3 | 0 | 13 | 13 | 3 | 0 | 0 | 0 |
| Gt-Gby | 21 | 3 | 3 | 1 | 13 | 13 | 3 | 0 | 0 | 0 |
| Gb-Gd | 13 | 2 | 0 | 2 | 11 | 2 | 0 | 0 | 0 | 0 |
| Gb-Gh | 25 | 5 | 3 | 5 | 21 | 13 | 3 | 2 | 1 | 0 |
| Gb-Ghl | 29 | 3 | 3 | 6 | 19 | 9 | 3 | 2 | 1 | 0 |
| Gb-Gm | 34 | 4 | 4 | 3 | 16 | 11 | 3 | 3 | 1 | 0 |
| Gb-Ghh | 31 | 5 | 3 | 5 | 22 | 14 | 4 | 2 | 1 | 0 |
| Gb-Gt | 21 | 3 | 2 | 1 | 13 | 13 | 3 | 0 | 0 | 0 |
| Gb-Gbk | 4 | 0 | 0 | 0 | 0 | 0 | 0 | 0 | 0 | 0 |
| Gb-Gby | 1 | 1 | 0 | 0 | 0 | 0 | 0 | 0 | 0 | 0 |
| Gbk-Gh | 25 | 6 | 2 | 5 | 22 | 12 | 3 | 2 | 1 | 0 |
| Gby-Gh | 25 | 5 | 2 | 5 | 21 | 13 | 3 | 2 | 1 | 0 |
| Gby-Gbk | 5 | 0 | 0 | 0 | 0 | 0 | 0 | 0 | 0 | 0 |

**Table S8B** The number of indels in different length between other subspecies plastomes

| **Species** | **1 bp** | **2 bp** | **3 bp** | **4 bp** | **5 bp** | **6 bp** | **7 bp** | **8 bp** | **9 bp** | **10 bp** |
| --- | --- | --- | --- | --- | --- | --- | --- | --- | --- | --- |
| *S.lycopersicum-S.bulbocastanum* | 48 | 14 | 9 | 9 | 8 | 13 | 12 | 2 | 7 | 1 |
| *S.tuberosum-S.bulbocastanum* | 24 | 5 | 6 | 3 | 0 | 2 | 3 | 3 | 3 | 2 |
| *S.tuberosum-S.lycopersicum* | 47 | 16 | 9 | 7 | 8 | 11 | 13 | 3 | 4 | 3 |
| *N.tabacum-N.tomentosiformis* | 53 | 21 | 12 | 9 | 10 | 8 | 3 | 3 | 11 | 3 |
| *N.sylvestris-N.tomentosiformis* | 52 | 21 | 13 | 9 | 10 | 8 | 3 | 3 | 11 | 3 |
| *N.sylvestris-N.tabacum* | 2 | 1 | 0 | 0 | 0 | 0 | 0 | 0 | 0 | 0 |
| *Oryza sativa indica-Oryza sativa japonica* | 99 | 12 | 3 | 2 | 3 | 3 | 1 | 0 | 0 | 0 |
| *O.biennis-O.argillicola* | 57 | 31 | 31 | 15 | 20 | 20 | 11 | 6 | 3 | 5 |
| *O.biennis-O.glazioviana* | 29 | 15 | 17 | 12 | 13 | 15 | 8 | 4 | 2 | 1 |
| *O.elata-O.parviflora* | 61 | 18 | 17 | 13 | 27 | 20 | 8 | 6 | 6 | 3 |
| *O.argillicola-O.parviflora* | 34 | 11 | 14 | 14 | 11 | 9 | 5 | 3 | 1 | 0 |
| *O.parviflora-O.glazioviana* | 67 | 21 | 21 | 13 | 24 | 18 | 8 | 3 | 3 | 2 |
| *O.argillicola-O.glazioviana* | 49 | 31 | 37 | 12 | 21 | 17 | 6 | 5 | 4 | 3 |
| *O.elata-O.glazioviana* | 25 | 12 | 19 | 8 | 18 | 20 | 5 | 3 | 4 | 1 |
| *O.argillicola-O.elata* | 48 | 22 | 34 | 13 | 27 | 24 | 8 | 5 | 4 | 4 |
| *O.biennis-O.parviflora* | 66 | 26 | 19 | 13 | 22 | 19 | 11 | 6 | 4 | 4 |
| *O.biennis-O.elata* | 21 | 8 | 5 | 7 | 7 | 3 | 3 | 2 | 2 | 0 |

Note: We compared intraspecies or inter-subspecies of *Acorus*, *Aethionema*, *Nicotiana*, *Oryza*, *Oenothera*, *Populas*, and *Solanaceae*. Here *S.* (*Solanaceae*), *N*. (*Nicotiana*), and *O.* (*Oenothera*) were listed.
