# Supplementary material for: Analysis of Complete Nucleotide Sequences of 12 Gossypium Chloroplast Genomes: Origin and Evolution of Allotetraploids
Source: PLoS One. 2012 Aug 2;7(8):e37128. doi: 10.1371/journal.pone.0037128 (PMC3411646; doi:10.1371/journal.pone.0037128)
Supplement: Table S9 — The estimated indel ratio between Gossypium chloroplast genomes. (DOC) [file pone.0037128.s012.doc]

**Table S9** The estimated indel ratio between *Gossypium* chloroplast genomes

|  | **Gaf A1** | **Ga A2** | **Gh AD1** | **Ghh AD1** | **Ghl AD1** | **Gb AD2** | **Gbk AD2** | **Gby AD2** | **Gt AD3** | **Gm AD4** | **Gd AD5** | **Gr D5** | **Gg D6** |
| --- | --- | --- | --- | --- | --- | --- | --- | --- | --- | --- | --- | --- | --- |
| Gaf A1 |  |  |  |  |  |  |  |  |  |  |  |  |  |
| Ga A2 | 50 |  |  |  |  |  |  |  |  |  |  |  |  |
| Gh AD1 | 90 | 98 |  |  |  |  |  |  |  |  |  |  |  |
| Ghh AD1 | 105 | 115 | 225 |  |  |  |  |  |  |  |  |  |  |
| Ghl AD1 | 87 | 96 | 275 | 400 |  |  |  |  |  |  |  |  |  |
| Gb AD2 | 129 | 131 | 140 | 179 | 135 |  |  |  |  |  |  |  |  |
| Gbk AD2 | 119 | 120 | 138 | 152 | 133 | 63 |  |  |  |  |  |  |  |
| Gby AD2 | 129 | 131 | 137 | 177 | 133 | - | 63 |  |  |  |  |  |  |
| Gt AD3 | 98 | 97 | 118 | 132 | 112 | 150 | 119 | 150 |  |  |  |  |  |
| Gm AD4 | 108 | 114 | 150 | 157 | 138 | 127 | 244 | 323 | 152 |  |  |  |  |
| Gd AD5 | 111 | 111 | 137 | 153 | 133 | 194 | 131 | 183 | 92 | 179 |  |  |  |
| Gr D5 | 34 | 33 | 33 | 34 | 33 | 37 | 36 | 37 | 35 | 35 | 36 |  |  |
| Gg D6 | 34 | 33 | 34 | 35 | 33 | 38 | 37 | 38 | 36 | 35 | 37 | 45 |  |

Note：Indel ratio was standardized to the number of indels between genomes per MYA based on whole genome indels (Table 2) and evolutionary distance (Table 5).
