# Supplementary material for: Analysis of Complete Nucleotide Sequences of 12 Gossypium Chloroplast Genomes: Origin and Evolution of Allotetraploids
Source: PLoS One. 2012 Aug 2;7(8):e37128. doi: 10.1371/journal.pone.0037128 (PMC3411646; doi:10.1371/journal.pone.0037128)
Supplement: Table S10 — Primers employed for sequencing. (DOC) [file pone.0037128.s013.doc]

**Table S10** Primers employed for sequencing

| **Primer** | **Genome position^a^** | **Forward** | **Reverse** |
| --- | --- | --- | --- |
| GCID2 | 1923–2178 | CTTGCATTTTTCATTGCAC | AATGCTCTGGTCAATCATTC |
| GCID14 | 37412–37653 | ACATGGCCATATGAGTTGAT | ATGCAAGGTAGAGGGATTTT |
| GCID22 | 64849–65078 | GCTCCTTCGTCTCAAAATC | GTGCTTAGCCCTTGAATCTA |
| GCID28 | 86780–87022 | ACATTGCTCTTTATGAGATGC | ATCTGGATCCAAAGAATCAG |
| GCID34 | 115977–116209 | CACGACCTTGAACAGACAC | TATGACCATCGAGGAACTTT |
| GCID78 | 130626–130828 | GGTTAGTTTCGACAATCCAG | GGATTCTTATTTTCCCCATC |
| CPS9 | 87112–100825 | CCCAACGAGTCGCACACTAAGCAT | GCAGGGTCAGGAACAACGATTCTC |
| CPS10 | 100667–114506 | CGACGGATGCTCCTATTACACTC | CCCAGTTGTTGCTGATACTCTC |
| CPS14 | 133522–148243 | CACTTTCTCTTTGCGGTATTCCACCC | CGTTGTTCCTGACCCTGCTTCACC |
| CPS15-2 | 146958–917 | GAATGGCAGAGGCAAATAGAGC | TTCCAGGCTGAACACAACATCC |

Note: ^a^Position located in chloroplast genome of *G. hirsutum*. The primers entitled “GCID” were used to screen cpDNA Fosmid clones and “CPS” were for PCR in IRa and IRb.
