# Supplementary material for: Analysis of Complete Nucleotide Sequences of 12 Gossypium Chloroplast Genomes: Origin and Evolution of Allotetraploids
Source: PLoS One. 2012 Aug 2;7(8):e37128. doi: 10.1371/journal.pone.0037128 (PMC3411646; doi:10.1371/journal.pone.0037128)
Supplement: Table S11 — Chloroplast genomes of species adopted in current study. (DOC) [file pone.0037128.s014.doc]

**Table S11** Chloroplast genomes of species adopted in current study

| **Classification/species** | **GenBank**  **Accession NO.** | **Reference** |
| --- | --- | --- |
| **Gymnosperm** |  |  |
| *Pinus thunbergii* | D17510 | Wakasugi et al. (1994) |
| **Basal angiosperms** |  |  |
| *Amborella trichopoda* | AJ506156 | Goremykin et al. (2003) |
| *Nuphar advena* | DQ354691 | Raubeson et al. (2007) |
| **Angiosperms** |  |  |
| **Monocots** |  |  |
| *Acorus calamus* | AJ879453 | Goremykin et al. (2005) |
| *Typha latifolia* | GU195652 | Guisinger et al. (2010) |
| *Zea mays* | X86563 | Maier et al. (1995) |
| *Triticum aestivum* | AB042240 | Ogihara et al. (2002) |
| **Dicots** |  |  |
| *Ranunculus macranthus* | DQ359689 | Raubeson et al. (2007) |
| *Aethionema grandiflorum* | AP009367 | Hosouchi et al. (2007) |
| *Barbarea verna* | AP009370 | Hosouchi et al. (2007) |
| *Arabidopsis thaliana* | AP000423 | Sato et al. (1999) |
| *Gossypium herbaceum* var. *africanum* | HQ325742 | Current study |
| *G*. *arboreum* | HQ325740 | Current study |
| *G. raimondii* | HQ325744 | Current study |
| *G*. *gossypioides* | HQ901195 | Current study |
| *G*. *hirsutum* | DQ345959 | Lee et al. (2006) |
| *G. hirsutum* race *hainansijimian* | HQ901197 | Current study |
| *G. hirsutum* race *lanceolatum* | HQ901196 | Current study |
| *G*. *barbadense* | AP009123 | Ibrahim et al. (2006) |
| *G. barbadense* cv. Zhonghai 7 | HQ901199 | Current study |
| *G*. *barbadense* race *yuanmou* | HQ901198 | Current study |
| *G*. *barbadense* race *kaiyuan* | HQ901200 | Current study |
| *G*. *tomentosum* | HQ325745 | Current study |
| *G*. *mustelinum* | HQ325743 | Current study |
| *G*. *darwinii* | HQ325741 | Current study |
